# A Non-enveloped Virus Hijacks Host Disaggregation Machinery to Translocate across the Endoplasmic Reticulum Membrane

**DOI:** 10.1371/journal.ppat.1005086

**Published:** 2015-08-05

**Authors:** Madhu Sudhan Ravindran, Parikshit Bagchi, Takamasa Inoue, Billy Tsai

**Affiliations:** Department of Cell and Developmental Biology, University of Michigan Medical School, Ann Arbor, Michigan, United States of America; National Cancer Institute, UNITED STATES

## Abstract

Mammalian cytosolic Hsp110 family, in concert with the Hsc70:J-protein complex, functions as a disaggregation machinery to rectify protein misfolding problems. Here we uncover a novel role of this machinery in driving membrane translocation during viral entry. The non-enveloped virus SV40 penetrates the endoplasmic reticulum (ER) membrane to reach the cytosol, a critical infection step. Combining biochemical, cell-based, and imaging approaches, we find that the Hsp110 family member Hsp105 associates with the ER membrane J-protein B14. Here Hsp105 cooperates with Hsc70 and extracts the membrane-penetrating SV40 into the cytosol, potentially by disassembling the membrane-embedded virus. Hence the energy provided by the Hsc70-dependent Hsp105 disaggregation machinery can be harnessed to catalyze a membrane translocation event.

## Author Summary

How non-enveloped viruses penetrate a host membrane to enter cells and cause disease remains an enigmatic step. To infect cells, the non-enveloped SV40 must transport across the ER membrane to reach the cytosol. In this study, we report that a cellular Hsp105-powered disaggregation machinery pulls SV40 into the cytosol, likely by uncoating the ER membrane-penetrating virus. Because this disaggregation machinery is thought to clarify cellular aggregated proteins, we propose that the force generated by this machinery can also be hijacked by a non-enveloped virus to propel its entry into the host.

## Introduction

Protein misfolding and aggregation compromise cellular integrity. Cells in turn deploy powerful molecular chaperones to promote protein folding, prevent aggregation, and in some instances, re-solubilize the aggregated toxic species to rectify these problems and maintain proper cellular function [1–3]. A cell’s ability to effectively mount a response to protein misfolding and aggregation despite acute or sustained environmental stresses has major implications in the development of protein conformational-based diseases [4,5].

The 110 kDa heat shock protein (Hsp110) family, including Hsp105, Apg1, and Apg2, are cytosolic chaperones that belong to the Hsp70 superfamily [6–10]. In addition to serving housekeeping roles during protein homeostasis, this protein family has been linked to wide ranging cellular processes including cell migration [11], spindle length control [12], and molecular scaffolding [13]. Importantly, as the Hsp110 family has also been implicated in many protein misfolding diseases, such as amyotrophic lateral sclerosis [14,15], prion disease [16], Alzheimer’s disease [17], cystic fibrosis [18], and polyglutamine disease [19,20], clarifying its precise mechanism of action in cells is paramount.

At the molecular level, Hsp110 acts as a nucleotide exchange factor (NEF) against Hsp70 and the constitutively expressed Hsc70 [7,8], which was used in this study. A NEF triggers nucleotide exchange of ADP-Hsc70, generating ATP-Hsc70 that displays a low affinity for its substrate [21]. This reaction reverses the effect of a J-protein, which uses its J-domain to stimulate the ATPase activity of ATP-Hsc70, forming ADP-Hsc70 that binds to its substrate with high affinity. Thus, a typical substrate-binding and release cycle by Hsc70 is coordinately regulated by a NEF and a J-protein.

Structurally, Hsp110 harbors an N-terminal ATPase domain similar to Hsc70, followed by a peptide-binding domain, an acidic loop, and a C-terminal helix domain thought to sub-serve a “holdase” function [6]. Strikingly, beyond simply acting as a NEF, reports suggest that Hsp110, in conjunction with the Hsc70:J-protein complex, can function as a disaggregase against model substrates [7,22–25]. However, whether Hsp110 and its chaperone activity acts on a physiologically relevant substrate as part of a cell’s protein quality control response, or is exploited to promote other unanticipated biological processes, is unclear. Here we demonstrate a novel and unexpected role of Hsp110 in driving membrane translocation of a virus.

To cause infection, the non-enveloped polyomavirus (PyV), typified by the classic simian PyV SV40, traffics from the host cell surface to the ER from where it penetrates the ER membrane to reach the cytosol [26–29]. In the cytosol, the virus moves into the nucleus to enable transcription and replication of the viral genome, causing lytic infection or cellular transformation. Our understanding of how SV40 is extracted into the cytosol from the ER is slowly unraveling.

Structurally, SV40 is composed of 360 copies of the major coat protein VP1 arranged as 72 pentamers, with each pentamer engaging either of the internal hydrophobic protein VP2 or VP3. The pentamers are assembled as a 45 nm-diameter icosahedral particle which in turn encapsulates its viral DNA genome [30,31]. Upon reaching the ER [32–34], the virus hijacks ER-resident isomerase and reductase that impart conformational changes to the viral particle to expose its hydrophobic proteins VP2 and VP3 without triggering massive disassembly [35–41]. These remodeling reactions generate a hydrophobic particle that binds to and integrates into the ER membrane [35,37,42]. However, the molecular mechanism by which the membrane-embedded intact hydrophobic virion is extracted into the cytosol and disassembled is not entirely clear.

A clue to unraveling this mystery emerged when three ER membrane J-proteins called DnaJB12 (B12), DnaJB14 (B14), and DnaJC18 (C18) were reported to support ER-to-cytosol translocation of SV40 and the related human BK PyV (BKV) [38]. Because the J-domain of B12, B14, and C18 orient towards the cytosol, we hypothesized that they recruit the cytosolic Hsc70 machinery to the ER membrane where it engages and ejects SV40 from the ER into the cytosol. In fact, we identified an Hsc70 co-chaperone called SGTA that forms part of a poorly-defined ER membrane-localized Hsc70 complex (which comprises of at least B14-Hsc70-SGTA) that supports extraction of PyV into the cytosol [43]. However, the precise molecular nature of this machinery as well as how SV40 is ultimately ejected into the cytosol from the ER is not completely defined. In this study, we identify the Hsp110 family member Hsp105 as a novel B14 binding partner. ER membrane juxtaposition of Hsp105 enables it to interact with and promote the extraction of membrane-embedded SV40 into the cytosol, thereby preparing the virus for infection. Our data support a scenario whereby Hsp105 synergizes with the B14-Hsc70-SGTA to extract SV40 into the cytosol, potentially by disassembling the virus.

## Results

### The cytosolic Hsp105 interacts with the ER membrane J-protein B14

**Fig 1:**
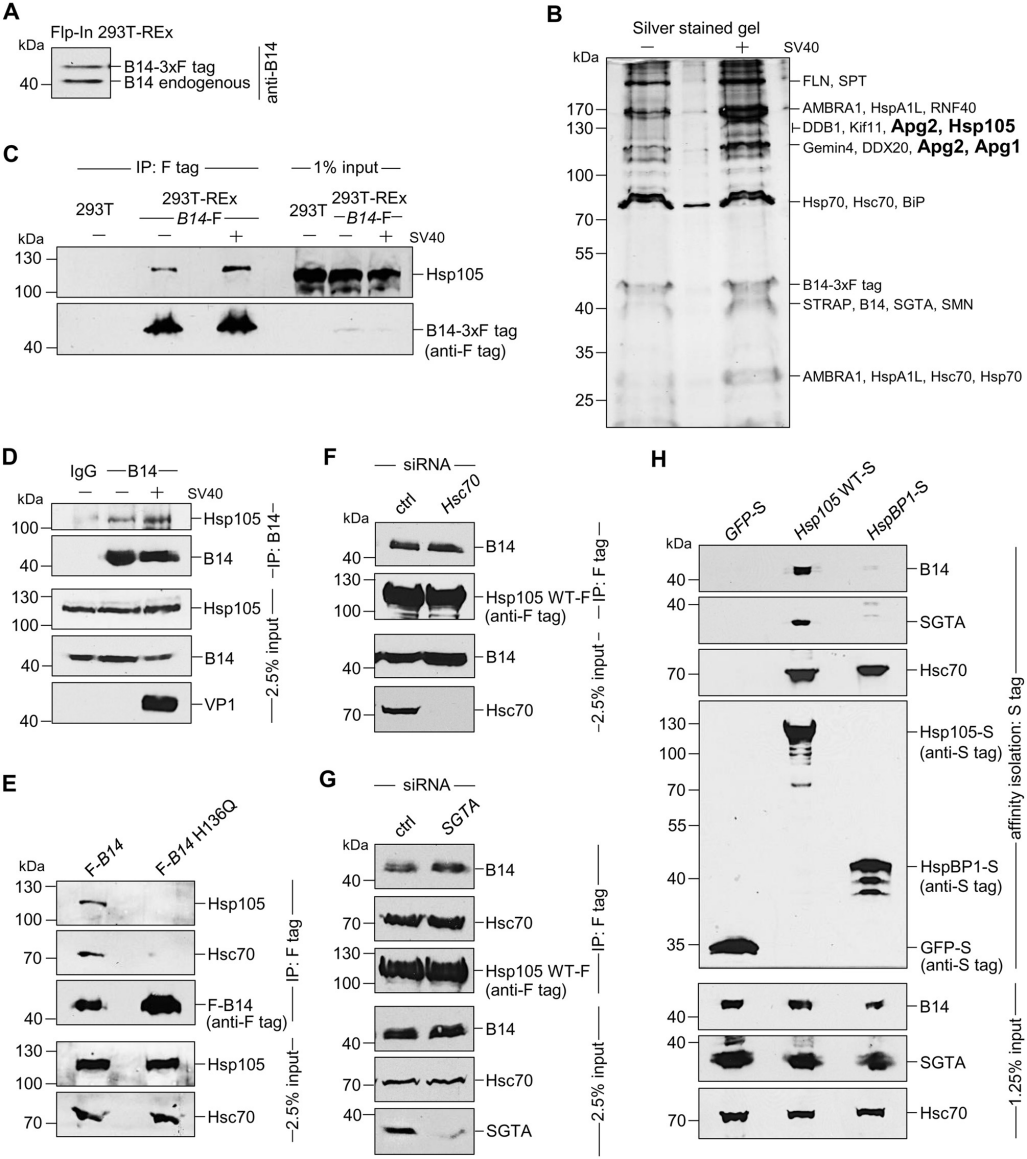
The cytosolic Hsp105 interacts with the ER membrane J-protein B14 **A**. Expression of B14-3xF and endogenous B14 in Flp-In 293 T-Rex cell lysates were analyzed by immunoblotting against B14. A corresponding molecular weight marker in kDa is shown on the left. **B**. B14-3xF was immunopurified from Flp-In 293 T-Rex cells infected with SV40 MOI ~50 (‘+’) or uninfected (‘-’). Bound proteins were eluted with 3x FLAG peptide, and the samples separated by SDS-PAGE followed by silver staining. Bands (indicated on the right) were excised and subjected to mass spectrometry analysis. Protein identities of the bands are listed on the right side of the gel. **C**. Samples in (B) were immunoblotted with the indicated antibodies. Uninfected HEK 293T cells not expressing B14-3xF were used as a control. **D**. CV-1 cells were cross-linked with DSP, lysed, the endogenous B14 immunoprecipitated, and the precipitated samples subjected to immunoblotting using the indicated antibodies. Where indicated, cells were infected with SV40. **E**. Cells expressing F-B14 or F-B14 H136Q were cross-linked, lysed, and the FLAG-tagged proteins immunoprecipitated followed by immunoblotting using the indicated antibodies. **F**. CV-1 cells treated with a control (ctrl) or *Hsc70* siRNA were transfected with *Hsp105* WT-F, and the FLAG-tagged protein immunoprecipitated followed by immunoblotting using the indicated antibodies. **G**. As in F, except *SGTA* siRNA was used. **H**. The S-tagged protein in CV-1 cells were affinity purified and immunoblotted using the indicated antibodies.

As B14 mediates SV40 ER membrane translocation [38], we reasoned that pinpointing B14’s interacting partners might reveal how the ER membrane-penetrating SV40 is extracted into the cytosol. Combining immunoaffinity purification and mass spectrometry, we identified the cytosolic co-chaperone SGTA as a B14 steady-state binding partner that is part of an Hsc70 complex which mobilizes SV40 from the ER to the cytosol [43]. However, the precise biochemical mechanism by which the B14-Hsc70-SGTA complex promotes SV40 cytosol entry is unclear. To characterize additional interaction partners of this complex, we asked if novel cellular factors are recruited to B14-Hsc70-SGTA during SV40 infection. To this end, we used a HEK 293T cell line stably expressing 3x-FLAG tagged (hereon FLAG-tag is abbreviated as ‘F’) B14 (B14-3xF) that expresses a similar level of B14-3xF as endogenous B14 (Fig 1A). This human cell line, used previously to identify SGTA as a B14-interacting partner [43], supports SV40 infection when supplemented with the cell-surface SV40 receptor ganglioside GM1.

B14-3xF in the whole cell extract (WCE) derived from cells infected with or without SV40 was immunopurified and eluted (see Methods). The eluted material was subjected to SDS-PAGE and silver staining. Distinct bands were excised and analyzed by mass spectrometry. Interestingly, some of the band intensity appeared higher in samples obtained from SV40-infected cells when compared to the uninfected sample (Fig 1B). Results from mass spectrometry revealed the presence of B14, SGTA, and Hsc70/Hsp70, as anticipated. While numerous additional proteins were also identified, we focused our attention on the Hsp110 family members (Hsp105, Apg1, and Apg2 in Fig 1B, highlighted in bold) because they are established Hsc70/Hsp70 interacting partners [7,8]. To validate our mass spectrometry results, samples in Fig 1B were initially immunoblotted using an antibody against Hsp105 (see Table 1 for list of all antibodies used in this study), which confirmed Hsp105 co-precipitated with B14-3xF (Fig 1C, first panel). A parental 293T cell that does not express B14-3xF was used as a negative control (Fig 1C, lane 1). These findings demonstrate that B14-3xF interacts with Hsp105. We found a modest increase in the level of Hsp105 that co-precipitated with B14-3xF in SV40-infected cells when compared to uninfected cells (Fig 1C, first panel, compare lane 3 to 2), suggesting that Hsp105 is likely recruited to B14 during infection. We also established endogenous Hsp105-B14 interaction in the simian CV-1 cells by co-immunoprecipitation, with the association moderately enhanced during SV40 infection (Fig 1D, first panel, compare lane 3 to 2). This experiment was performed in the presence of the membrane permeable, amine-reactive, and thiol-cleavable crosslinker DSP (dithiobis(succinimidyl proprionate)) in order to stabilize transient or weak protein-protein interactions. CV-1 cells were analyzed because they are the normal permissive cells used to study SV40 infection. All studies hereafter were performed in this cell line unless otherwise indicated. We attempted but were not able to establish an interaction between B14 and Apg1/Apg2, nor with other proteins identified in the mass spectrometry results. For these reasons, we focused our efforts on Hsp105 in the rest of this study.

**Table 1:** Antibodies used in this study.

antibody	dilution	use	product details
Rabbit polyclonal anti-Hsp105	1:2500	IB	Santa Cruz sc6241
Rabbit polyclonal anti-Hsc70	1:2500	IB	Pierce PA5-27337
Rabbit polyclonal anti-B14	1:3000, 1:1000, 2.5 μg	IB, IF, IP	Proteintech 16501-1-AP
Rat monoclonal anti-BAP31	1:500	IF	Pierce MA3-002
Rabbit polyclonal anti-SGTA	1:3000	IB	Proteintech PTG11019-2-AP
Mouse monoclonal anti- SV40 large TAg	1:100	IF	Santa Cruz sc147
Mouse monoclonal anti-VP1	1:2000, 1:500, ~2 μg	IB, IF, IP	Walter Scott (Univ. of Miami)
Rabbit polyclonal anti-VP2/3	1:500	IF	Abcam ab53983
Mouse monoclonal anti-PDI	1:10000	IB	Abcam ab2792
Rabbit polyclonal anti-Hsp90	1:3000	IB	Santa Cruz sc7947
Rabbit polyclonal anti-S tag	1:2000, 1:100	IB, IF	Abcam ab18588
Rabbit polyclonal anti-FLAG tag	1:5000, 1:1000	IB, IF	Sigma F7425
Goat anti-rabbit (HRP conjugated)	1:3000	IB	Sigma A4914
Goat anti-mouse (HRP conjugated)	1:3000	IB	Sigma A4416
Goat polyclonal anti-rabbit (Alexa flour 488)	1:1000	IF	Life technologies A11008
Donkey polyclonal anti-rat (Alexa flour 594)	1:500	IF	Life technologies A21209
Goat polyclonal anti-mouse (Alexa flour 350)	1:100	IF	Life technologies A11045
Goat polyclonal anti-mouse (Alexa flour 488)	1:1000	IF	Life technologies A11029
Goat polyclonal anti-mouse (Alexa flour 594)	1:1000	IF	Life technologies A11032

To assess if the B14-Hsp105 interaction requires an intact B14 J-domain, we used a B14 J-domain mutant (B14 H136Q) that was previously shown to be defective in Hsc70 binding [44]. Precipitation of transiently transfected FLAG-tagged B14 (F-B14) or H136Q B14 (F-B14 H136Q) in CV-1 cells revealed that the mutant does not interact with endogenous Hsp105 or Hsc70 (Fig 1E, first and second panels), even though more mutant protein was precipitated. This result suggests that B14’s interaction with Hsp105 requires an intact J-domain, but it does not indicate the interaction is direct or mediated by Hsc70. To test this, we depleted Hsc70 from CV-1 cells (using an siRNA directed specifically against *Hsc70*) and found that depletion of Hsc70 did not impair binding between endogenous B14 and transiently transfected FLAG-tagged wild-type (WT) *Hsp105* (*Hsp105* WT-F) (Fig 1F, first panel; see Table 2 for list of all primers used in this study). These observations suggest that B14 utilizes its J-domain to bind to Hsp105 directly, although we cannot rule out the possibility that the related Hsp70 would provide the physical link between B14 and Hsp105 when Hsc70 is absent. Knockdown of SGTA also did not affect Hsp105 WT-F’s interaction with endogenous B14 or Hsc70 (Fig 1G, first and second panels), indicating that this co-chaperone unlikely controls Hsp105’s ability to complex with B14 and Hsc70. To further evaluate if the Hsp105 interaction with B14-Hsc70-SGTA complex was specific, we transiently transfected S-tagged WT Hsp105 (Hsp105 WT-S) or a cytosolic non-Hsp110 family NEF called HspBP1 (HspBP1-S). Whereas endogenous Hsc70 was pulled down by precipitating either Hsp105 WT-S or HspBP1-S (Fig 1H, third panel, lanes 2 and 3), only precipitation of Hsp105 WT-S pulled down endogenous B14 and SGTA (Fig 1H, first and second panels). We conclude that Hsp105 is specifically recruited to the ER membrane by anchoring to B14.

**Table 2:** Primers used in this study.

gene	primers
***Hsp105* WT-S**	
first PCR	Fwd 5’-TATATCTCGAGATGTCGGTGGTGGG-3’
	Rev 5’-CGAATTTAGCAGCAGCGGTTTCTTTGTCCAAGTCCATATTAACAG-3’
second PCR	Fwd same primer used for first PCR
	Rev 5’-ATACGCGGCCGCTCAGCTGTCCATGTGCTGGCGTTCGAATTTAGCAGCGG-3’
***Hsp105* WT-F**	
first PCR	Fwd 5’-TAATACGACTCACTATAGGG-3’
	Rev 5’-GTCCAAGTCCATATTAACAGAAT-3’
siRNA resistant	Fwd 5’-ACAAATTACTGCTATGCTGCTGACTAAGCTGAAGGAAACTGC-3’
	Rev 5’-GCAGCATAGCAGTAATTTGTTCCACACTAAATAGATGTTCTTC-3’
second PCR	Fwd same primer used for first PCR
	Rev 5’-ATATAGATCTCTACTTGTCATCGTCGTCCTTGTAGTCGTCCAAGTCCATATTAACAGAAT-3’
*Hsp105* NE*-F	
inverse PCR	Fwd 5’-TTTAGCATCATTCCTTTCTTTTTCCAATTTATCTTGCATTATCATCTTACCCTC-3’
	Rev 5’-TACGCAGTTGCCGAATATGTGTATGAGTTCAGAGACAAGCTG-3’
normal PCR	Fwd 5’-CTGACTCGAGATGTCGGTGGTGGGGTTGGACGTGCTGTCGCAGAGCTGCTACATCG-3’
	Rev 5’-TTTAGCATCATTCCTTTCTTTTTCC-3’
*Hsp105* G*-F	
normal PCR	Fwd 5’-CTGACTCGAGATGTCGGTGGTGGGGTTGGACGTGCTGTCGCAGAGCTGCTACATCGCGG-3’
	Rev 5’-TTTAGCATCATTCCTTTCTTTTTCC-3’
***HspBP1*-S**	
cloning	Fwd 5’-CCACCTTCTCCCTCTAACAC-3’
	Rev 5’-TCACCGATCCATGCTGTCGTC-3’
subcloning	Fwd 5’-ATATCTCGAGCCACCATGTCAGACGAAGGCTCAAG-3’
	Rev 5’-ATATGGATCCCCGATCCATGCTGTCGTCCG-3’
***Hsc70*-S**	
subcloning	Fwd 5’-ATATCTCGAGGCAACCATGTCCAAGGGACCTGCAGTTG-3’
	Rev 5’-TATAGGATCCATCAACCTCTTCAATGGTGG-3’

### Hsp105 is essential for polyomavirus infection

**Fig 2:**
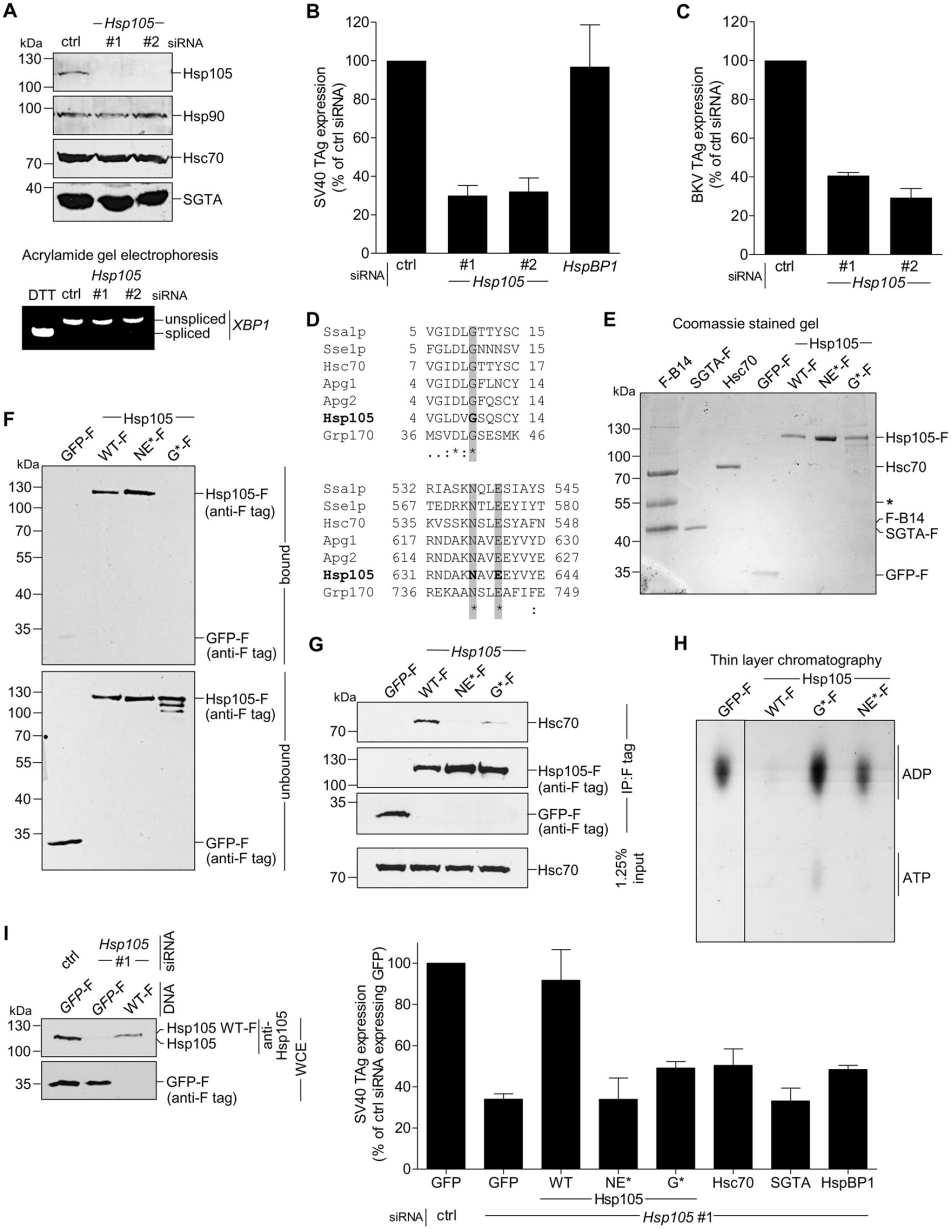
Hsp105 is essential for polyomavirus infection. **A**. CV-1 cells were transfected with a ctrl siRNA, or *Hsp105* siRNA #1 or #2 for 24 h, and the resulting WCE were immunoblotted with the indicated antibodies (top panel) or RT-PCR analysis was performed to observe the *XBP1* splicing (bottom panel). Cells treated with DTT were used as a positive control. **B**. Cells in (A) were infected with SV40 (MOI ~0.5) for 24 h, fixed, and immunostained against SV40 large T antigen (TAg). Infection was scored using immunofluorescence microscopy (counting >1000 cells for each condition). Data are normalized to the ctrl siRNA. Values represent the mean ± SD (n≥3). **C**. As in (B), except cells were infected with BKV for 40 h before immunostaining for BKV TAg. **D**. Multiple sequence alignment of Hsp70 and Hsp110 family proteins from yeast and humans. Only the relevant sequences are shown. The highlighted regions indicate the amino acid(s) that were altered to generate the Hsp105 mutants (see Methods). **E**. The indicated F-tagged proteins were purified from 293T cells, and their purity analyzed by SDS-PAGE followed by staining with Brilliant Blue R250. Hsc70 was obtained from commercial source (see Methods). The asterisk indicates an antibody heavy chain band. **F**. Purified proteins in (E) were incubated with ATP conjugated-agarose beads. Unbound and bound proteins were analyzed by immunoblotting using a FLAG antibody. **G**. CV-1 cells expressing the indicated F-tagged proteins were immunoprecipitated, and the eluted samples were analyzed by immunoblotting. **H**. Thin layer chromatography was used to determine the level of radiolabeled ADP that remain bound to Hsc70 after the indicated purified protein was incubated with radiolabeled ADP-Hsc70. The black line indicates that an intervening lane has been spliced out of the same film. **I**. CV-1 cells were reverse transfected with ctrl or *Hsp105* siRNA #1 for 24 h prior to transfection with the indicated tagged constructs for 24 h. Cells were then infected with SV40 (MOI ~0.5) for 24 h, fixed, and stained with anti-FLAG/S and anti-TAg antibodies. The percentage of TAg positive cells were counted only in cells expressing the indicated tagged protein by immunofluorescence microscopy (right graph). Values represent mean ± SD (n≥3). The protein expression levels of endogenous Hsp105, as well as transfected Hsp105 WT-F and GFP-F, in cell extracts derived from control and Hsp105-depleted cells (transfected with either *GFP*-F or *Hsp105* WT-F) are shown (left panels). The three lanes in the immunoblot correspond to the first three bars in the right graph.

To test whether Hsp105 is required for SV40 infection, CV-1 cells were transfected with either of two distinct siRNAs targeted against *Hsp105* (*Hsp105* #1 and #2), or a negative control siRNA (ctrl) (see Table 3 for list of all siRNAs used in this study). Immunoblotting of the resulting WCE demonstrated that the *Hsp105*-specific siRNAs efficiently knocked down endogenous Hsp105, without affecting the levels of the other cytosolic chaperones (Fig 2A, top panel) and without triggering *XBP1* splicing (see Table 4; Fig 2A, bottom panel), a sensitive readout of cellular ER stress induction. Under the knockdown condition, we evaluated SV40 infection by scoring for presence of the virally encoded large T antigen (TAg) in the host nucleus by immunostaining. *Hsp105* knockdown inhibited SV40 infection by 70–75% compared to control siRNA or a siRNA directed against *HspBP1* (Fig 2B). A similar infection block was also observed for BKV when *Hsp105* is knocked down (Fig 2C). Likewise, depletion of *Hsp105* in the simian BSC-1 cells (S1A Fig) reduced SV40 infection by 60% (S1B Fig). The residual infection found when *Hsp105* is down-regulated could be due to presence of other Hsp110 family members such as Apg1 or Apg2, or in the case of BSC-1 cells, the incomplete knockdown of Hsp105.

**Table 3:** siRNA used in this study.

siRNA	sequence (sense)	sequence (antisense)
***Hsp105* #1**	5’-GAGCAGAUAACAGCCAUGUUGUUGA-3’	5’-UCAACAACAUGGCUGUUAUCUGCUC-3’
***Hsp105* #2**	5’-GAAGGAGAGGACCAAGCUAAACAUU-3’	5’-AAUGUUUAGCUUGGUCCUCUCCUUC-3’
***SGTA***	5’-CAGCCUACAGCAAACUCGGCAACUA-3’	5’-UAGUUGCCGAGUUUGCUGUAGGCUG-3’
***Hsc70***	5’-GACCUUCACUACCUAUUCU-3’	5’-UGUUTUGGTUGTGUUGGTC-3’
***HspBP1***	5’-CAGGAAUGCUGAUUUGACCUUGAGC-3’	5’-GCUCAAGGUCAAAUCAGCAUUCCUG-3’
**control**	All-star negative (Qiagen catalogue: 1027281)	

**Table 4:** siRNA used in this study.

Gene	Forward	reverse
***XBP1***	5’-GAATGAAGTGAGGCCAGTGG-3’	5’-GGGGCTTGGTATATATGTGG-3’

As previous studies reported that the Hsp110 family acts as a NEF against Hsc70/Hsp70 [7,25,45], we asked if Hsp105’s nucleotide exchange activity is important in supporting SV40 infection. For this, we generated two Hsp105 mutants based on previous characterization of mammalian Hsp110. A mutant Apg2, referred to as the N619Y/E622A variant, is defective in its nucleotide exchange activity because it cannot interact with Hsc70 [24], consistent with a crystal structure of the yeast homolog demonstrating that these two residues are positioned at the interface required for interaction with Hsc70 [9,46]. Because N619 and E622 are highly conserved residues in the Hsp70 family/superfamily (Fig 2D, bottom panel), we generated the corresponding mutant (N636Y/E639A) in Hsp105 with FLAG tag (*Hsp105* NE*-F). In the second mutant, we took advantage of insights from studies on Grp170, the ER-resident Hsp110 family NEF [47–50]. In Grp170, mutating glycine at position 41 to leucine located within the ATP-binding pocket at its N-terminal ATPase domain renders the G41L mutant defective in its nucleotide exchange activity and impairs binding to the ER-resident Hsp70 BiP [51]. Since G41 is also conserved among Hsp70 family/superfamily (Fig 2D, top panel), we generated the corresponding mutant (G9L) in Hsp105 with FLAG tag (*Hsp105* G*-F).

We first assessed the behavior of Hsp105 NE*-F and G*-F. To act as a NEF against Hsc70, Hsp105 must bind to ATP, engage Hsc70, and then stimulate nucleotide release from Hsc70 [48]. Accordingly, we purified FLAG-tagged proteins from transfected 293T cells (Fig 2E). To determine the ATP-binding affinity of the Hsp105 mutants, purified proteins were incubated with ATP-conjugated agarose beads and eluted samples were analyzed by immunoblot. As predicted, Hsp105 WT-F and NE*-F but not G*-F bind to ATP (Fig 2F, first panel, compare lanes 2 and 3 to 4). By co-immunoprecipitation, neither Hsp105 NE*-F nor G*-F exhibited any significant binding affinity for Hsc70 (Fig 2G, first panel). Thus, while Hsp105 NE*-F binds to ATP, it cannot interact with Hsc70 due to mutations at the Hsc70 binding interface. By contrast, Hsp105 G*-F does not bind to ATP, likely leading to a defective ATPase activity essential for its interaction with Hsc70.

To evaluate the nucleotide exchange activity of Hsp105 mutants, we used a radioactive nucleotide release assay. Briefly, Hsc70 was preloaded with labeled nucleotide [α-^32^P]ATP which spontaneously hydrolyzes to generate [α-^32^P]ADP-Hsc70. Individual FLAG tagged protein along with unlabeled ATP was added to [α-^32^P]ADP-Hsc70 to induce nucleotide release. The amount of labeled ADP that remain bound to Hsc70 was evaluated by thin layer chromatography. Using this assay, we found that only Hsp105 WT-F but not the Hsp105 mutants triggered [α-^32^P]ADP release from Hsc70 (Fig 2H, compare lane 2 to 3 and 4), demonstrating that both Hsp105 mutants are indeed defective in their nucleotide exchange activity.

We performed knockdown followed by rescue experiments to probe the requirement of Hsp105’s nucleotide exchange activity during SV40 infection. Cells were initially transfected with control or *Hsp105* #1 siRNA, followed by transfection with *GFP*-F or an siRNA-resistant *Hsp105* WT-F, NE*-F, or G*-F. Cells were subsequently infected, fixed, and scored for the presence of TAg only in FLAG-expressing cells. Importantly, when compared to expressing the control GFP-F, only re-expressing Hsp105 WT-F but not NE*-F or G*-F under the *Hsp105* knockdown condition restored infection (Fig 2I, right graph). As controls, expressing Hsc70, SGTA, or HspBP1 when *Hsp105* is down-regulated did not significantly restore infection. We conclude that the nucleotide exchange activity of Hsp105 is required to promote successful SV40 infection. We note that the expression level of siRNA resistant Hsp105 WT-F in a cell extract derived from the total pool of Hsp105-depleted cells is less compared to the level of endogenous Hsp105 in control cells (Fig 2I, left first panel, compare lane 3 to 1). Given that the transfection efficiency in CV-1 cells is low (~20%), the re-expressed Hsp105 level is likely comparable to endogenous Hsp105. This observation is consistent with our finding that SV40 infection is only restored (but not enhanced) in Hsp105-reexpressed cells when compared to control cells.

### Hsp105 is indispensable for SV40 cytosol arrival

**Fig 3:**
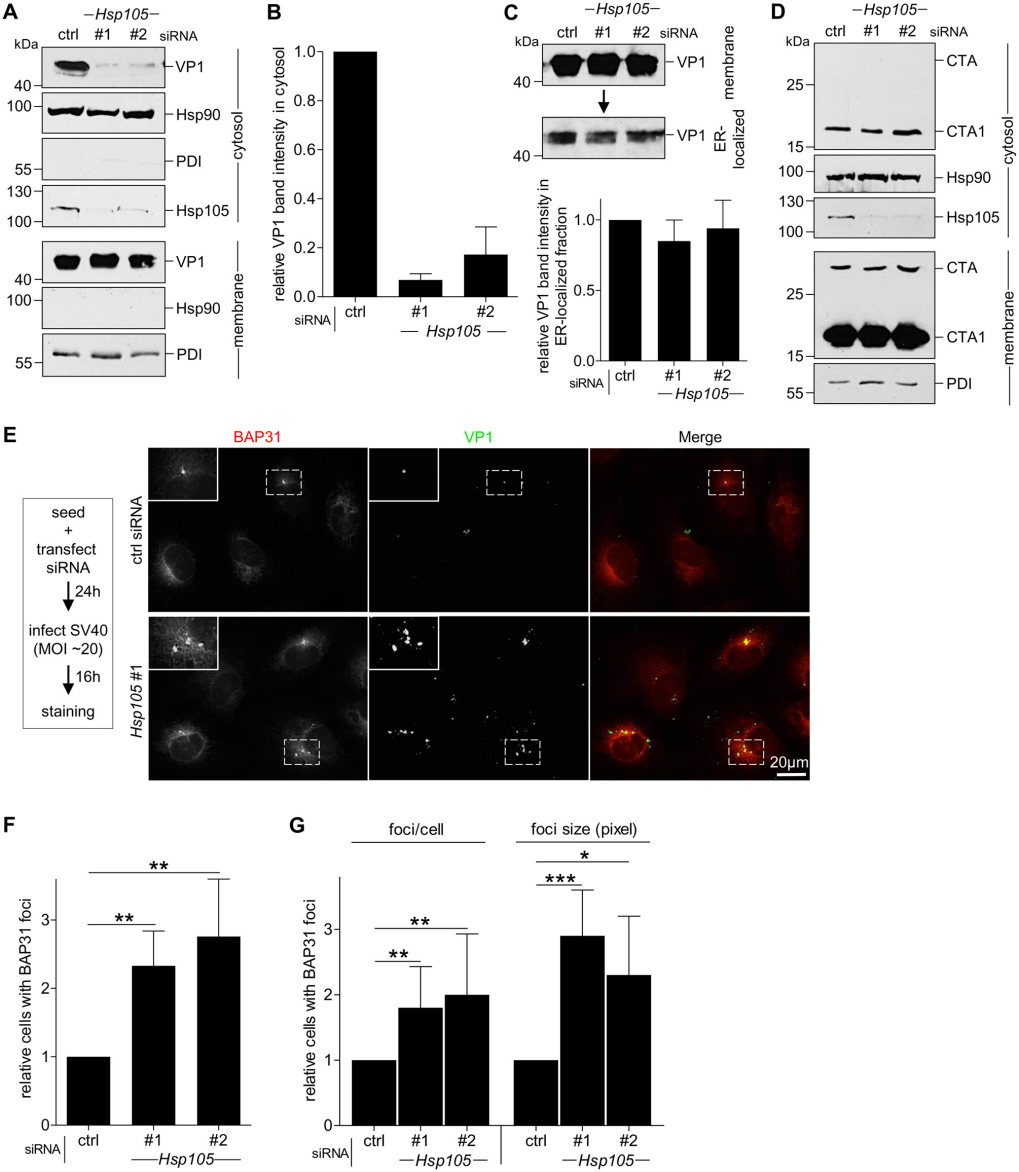
Hsp105 is indispensable for SV40 cytosol arrival. **A**. CV-1 cells transfected with the indicated siRNAs for 24 h were incubated with SV40 (MOI ~5), harvested 12 hpi, and processed according to the semi-permeabilized cytosol arrival assay (see Methods). Hsp90 and PDI serve as markers for the cytosol and membrane fraction, respectively. **B**. Relative VP1 band intensities in the cytosol fraction in (A) were quantified. Data are normalized to ctrl siRNA. Values represent the mean ± SD (n = 3). **C**. Membrane fraction in (A) was solubilized in a buffer containing 1% Triton X-100. After centrifugation, the extracted material containing ER-localized SV40 was analyzed by immunoblotting with VP1 antibodies (top panel). Relative VP1 band intensities in the ER-localized fraction were quantified as in (B) (bottom panel). **D**. As in (A), except cells were treated with 10 nM cholera toxin for 90 min before harvesting and analyzing using the indicated antibodies. **E**. CV-1 cells were transfected with either ctrl or *Hsp105* siRNA #1. After 24 h, cells were infected with SV40 (MOI ~20) for 16 h. Cells were then fixed, stained, and analyzed by immunofluorescence microscopy. The experimental set-up is depicted on the left side of the figure. The insert shows a 2x enlarged area of the dotted box. Scale bar, 20 μm. **F**. The siRNA-transfected cells were scored for the presence of at least one BAP31-positive focus in each cell, and the values normalized to the ctrl siRNA. The bar graphs represent mean values ± SD (n≥3). ** *p* <0.01. **G**. Results in (F) were further assessed by quantifying the number of BAP31 foci/cell under control and knockdown conditions, or the size of the BAP31 foci based on the measured area (in pixels) using the ImageJ software. * *p* <0.05, ** *p* <0.01, *** *p* <0.001.

As Hsp105 localizes to the ER membrane and is important in SV40 infection, we hypothesized that it might promote the extraction of SV40 into the cytosol from the ER membrane. To test this, we monitored arrival of SV40 into the cytosol from the ER membrane using a semi-permeabilized cytosol arrival assay established previously [37,39]. In this assay, siRNA transfected CV-1 cells were harvested post-infection and treated with a low concentration of digitonin to semi-permeabilize the plasma membrane without affecting internal membranes. Subsequent centrifugation generates two fractions, a supernatant fraction that harbors cytosolic proteins and virus that reaches the cytosol (referred as “cytosolic” fraction), and a pellet fraction that contains membranes including the ER, as well as associated viral particles (referred as “membrane” fraction). The cytosolic Hsp90 was found predominantly in the cytosolic fraction (Fig 3A, second panel), while the ER marker protein disulfide isomerase (PDI) was found exclusively in the membrane fraction (Fig 3A, seventh panel), verifying the integrity of the fractionation procedure. Importantly, using this assay, silencing *Hsp105* by either siRNA (Fig 3A, fourth panel) markedly decreased the VP1 level in the cytosol (Fig 3A, first panel, compare lanes 2 and 3 to 1; the VP1 band intensity is quantified in Fig 3B). These data indicate that Hsp105 exerts an important role in promoting cytosol arrival of SV40 from the ER. The residual VP1 observed in the cytosol fraction when *Hsp105* is knocked down is consistent with the low infection observed when this chaperone is down-regulated (Fig 2B).

To test if trafficking of SV40 from the cell surface to the ER is controlled by Hsp105, we isolated ER-localized SV40 (which includes viral particles in the ER lumen and those integrated into the ER bilayer during membrane penetration) using a previously established, Triton X-100 extraction protocol [39,43]. Using this strategy, we found that the level of ER-localized SV40 was unperturbed by down-regulating *Hsp105* (Fig 3C, top panel; the VP1 band intensity is quantified in bottom panel). Hence, the block in cytosol arrival of SV40 when *Hsp105* is knocked down (Fig 3A and 3B) cannot be attributed to a disruption in trafficking of the viral particle from the plasma membrane to the ER. This finding strengthens our proposal that Hsp105 regulates cytosol arrival of SV40 from the ER.

Another toxic agent that uses the ER-to-cytosol membrane translocation pathway to cause disease is cholera toxin (CT) [52,53]. However, in this case, knockdown of *Hsp105* (Fig 3D, third panel) did not affect cytosol arrival of CT’s catalytic CTA1 subunit from the ER (Fig 3D, first panel). This finding not only indicates that Hsp105 specifically controls cytosol entry of SV40 and not another toxic agent, but also suggests that silencing *Hsp105* did not globally disable cytosol entry machineries from the ER.

In addition to the cell-based approach, we used imaging strategies to further support the idea that Hsp105 promotes extraction of ER-localized SV40 into the cytosol. In the ER, SV40 accumulates in discrete foci where specific ER membrane proteins important for virus infection are also recruited [37,43,54]. An example of the SV40-induced foci is presented in S2 Fig, in which infected cells were immunostained for the viral proteins VP1 and VP2/3 (first and second row, white arrows); these foci colocalize with the ER membrane protein BAP31 which is also essential in virus infection [37]. As all BAP31 foci colocalize with ER-localized SV40, BAP31 foci serves as a convenient marker for SV40-containing foci. Under higher magnification, the intensity surface plots of several representative foci indicate that each focus contains several discrete spots, likely corresponding to multimeric viral particles (S3 Fig, surface plots on the right).

We hypothesize that these SV40-induced foci represent the cytosol entry site for the virus based on several lines of evidence. First, the VP2/3-exposed, membrane penetration-competent SV40 colocalizes with the foci [54] (S2 Fig, second row). Second, specific ER membrane proteins that facilitate SV40 membrane penetration, including B14/B12 (third and fourth rows) and BAP31 (all rows), are recruited to the foci [37,43]. Third, foci formation kinetics temporally parallels SV40 cytosol entry [43]. And fourth, SV40 mutants that cannot penetrate the ER membrane to access the cytosol fail to induce foci [37,54].

We reasoned that if the foci harboring SV40 represent the site from where ER-localized virus enters the cytosol, depleting Hsp105 (which prevents SV40 extraction into the cytosol, Fig 3A and 3B) should trap the virus in the ER membrane, thereby enhancing the foci structure. To evaluate this, cells transfected with either a control or *Hsp105* #1 siRNA were infected with SV40 (Fig 3E). When compared to control, silencing *Hsp105* increased the number of cells with at least one BAP31-positive foci by approximately 2-fold (Fig 3E; quantified in Fig 3F); a similar 2-fold increase was also observed using *Hsp105* siRNA #2 (Fig 3F). Additionally, in the *Hsp105* knockdown cells, there were more foci per cell and the size of the foci in the knockdown cells appears larger when compared to control (Fig 3E, see insert for 2x enlarged dotted box; quantified in Fig 3G). These data suggest that depleting Hsp105 entraps SV40 in the ER because it cannot be extracted into the cytosol, consequently enhancing foci formation. These imaging approaches were consistent with the cell-based studies, strongly suggesting that Hsp105 plays a key role in extracting SV40 from the ER into the cytosol.

### Hsp105 overexpression enhances SV40 extraction into the cytosol

**Fig 4:**
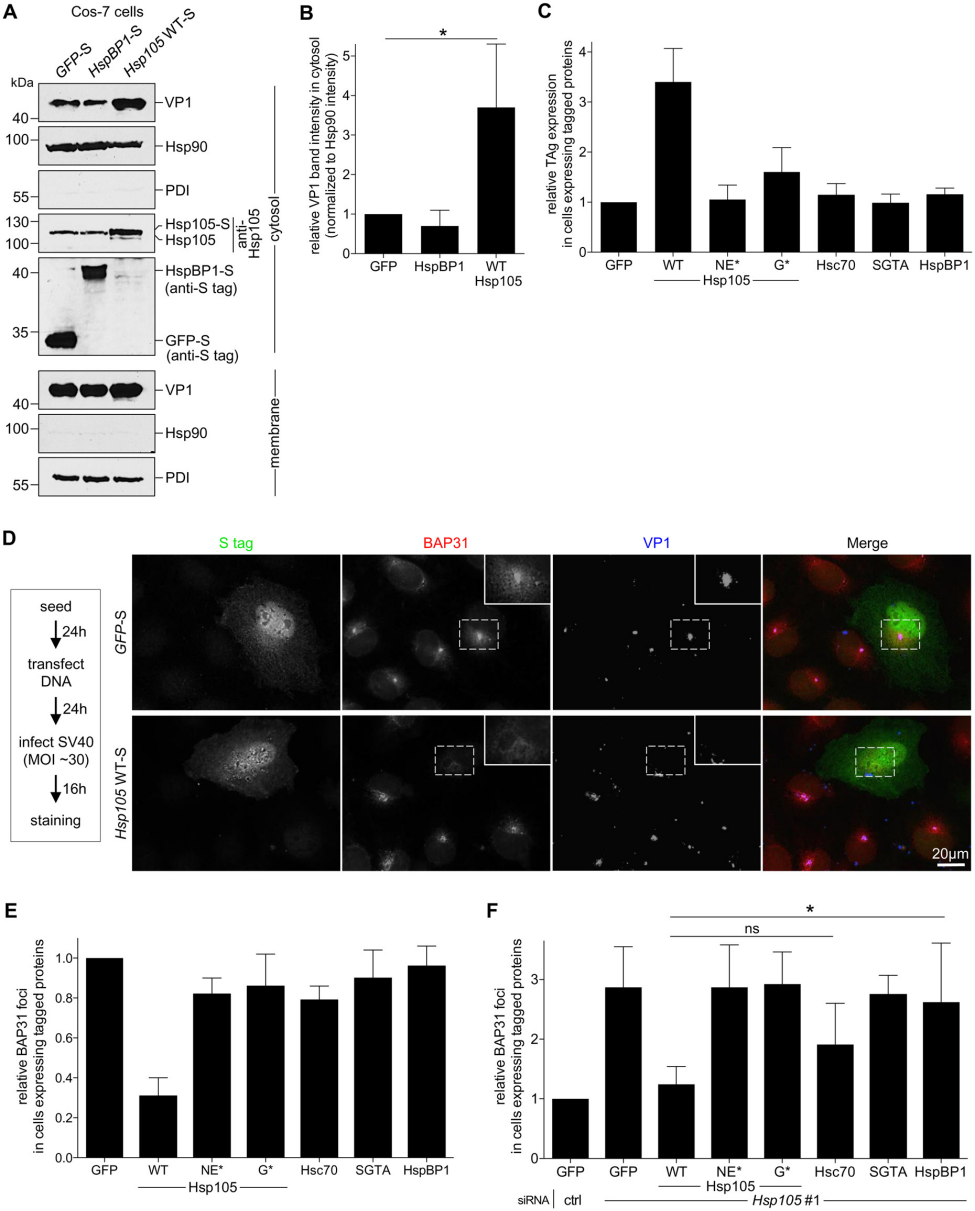
Hsp105 overexpression enhances SV40 extraction into the cytosol. **A**. COS-7 cells expressing the indicated S-tagged construct were infected with SV40 (MOI ~5). 12 hpi, cells were processed as in Fig 3A, and the samples immunoblotted using the indicated antibodies. **B**. VP1 band intensities of the cytosol-localized SV40 in (A) were quantified and normalized against the Hsp90 band intensity. Values represent the mean ± SD (n≥3). * *p* <0.05. **C**. Cells expressing the indicated F- or S-tagged construct are scored for the TAg expression after 24 hpi (MOI ~0.5), and the values normalized to GFP. The bar graphs represent mean values ± SD (n≥3). **D**. CV-1 cells expressing GFP-S or Hsp105 WT-S were infected with SV40 (MOI ~30) for 16 h. Cells were fixed, stained with BAP31 and VP1 antibodies, and imaged as in Fig 3E. The experimental set-up is depicted on the left side of the figure. In the first column, a representative cell expressing GFP-S (top row) or Hsp105 WT-S (second row) amongst cells not expressing this protein is shown. The insert shows a 2x enlarged area of the dotted box. Scale bar, 20 μm. **E**. Cells expressing the indicated tagged construct are scored for the presence of at least one BAP31-positive focus in each cell, and the values normalized to GFP. The bar graphs represent mean values ± SD (n≥3). **F**. Cells transfected with either ctrl or *Hsp105* siRNA #1 were subsequently transfected with the indicated tagged construct, and BAP31 foci were quantified as in (E). * *p* <0.05.

We used a gain-of-function strategy to assess Hsp105’s role in extracting SV40 into the cytosol. The CV-1 derived COS-7 cells were transfected with *GFP*-S, *HspBP1*-S, or *Hsp105* WT-S, and subjected to the cytosol arrival assay as described above. COS-7 cells were used because of their ability to support a high DNA transfection efficiency required for this experiment. We found that over-expressing Hsp105 WT-S (Fig 4A, fourth panel, compare lane 3 to 2 and 1) but not HspBP1-S stimulated SV40 arrival to the cytosol (Fig 4A, first panel; the VP1 band intensity is quantified in Fig 4B). These findings demonstrate that Hsp105 stimulates SV40 extraction into the cytosol. Not surprisingly, Hsp105 WT overexpression also enhanced SV40 infection (Fig 4C, compare second to first bar). This stimulation requires Hsp105’s nucleotide exchange activity as overexpressing Hsp105 NE*-F or G*-F did not robustly enhance infection (Fig 4C, compare third and fourth bars to second bar), and is specific because overexpressing neither Hsc70, SGTA, nor HspBP1 stimulated infection.

Again we used an imaging approach to further strengthen the idea that Hsp105 overexpression promotes extraction of ER-localized SV40 into the cytosol. We reasoned that, if the foci harboring SV40 represent the site from where ER-localized virus enters the cytosol, overexpressing Hsp105 (which stimulates virus extraction into the cytosol, Fig 4A and 4B) should correspondingly decrease the virus-induced foci. To test this, CV-1 cells expressing Hsp105 WT-S or the control GFP-S were infected with SV40, fixed, and immunostained (Fig 4D). Strikingly, whereas VP1-positive foci that colocalize with the BAP31 foci are found in GFP-S expressing cells (Fig 4D, first row, see insert for 2x enlarged dotted box), a dramatic decrease in these foci was observed in cells overexpressing Hsp105-S (Fig 4D, second row). We quantified these effects by scoring for presence of BAP31-positive foci in cells expressing GFP-S or Hsp105 WT-S; in our quantification, any cell that displays at least one BAP31-positive focus is scored positive. Our analyses revealed that overexpressing tagged Hsp105 decreased the number of cells containing at least one BAP31-positive focus by approximately 3-fold (Fig 4E, first two bar graphs), suggesting that overexpressing Hsp105 impairs foci formation. As Hsp105 overexpression also stimulates SV40 cytosol arrival (Fig 4A and 4B) and infection (Fig 4C), the ability of overexpressed Hsp105 to decrease foci formation likely reflects efficient Hsp105-dependent extraction of SV40 into the cytosol essential for infection.

To ascertain if Hsp105’s nucleotide exchange activity is necessary to decrease foci formation, cells were transfected with either *Hsp105* NE*-F or G*-F. Under these conditions, no significant reduction in the number of cells containing at least one BAP31-positive foci was found (Fig 4E, compare third and fourth bar graphs to second), suggesting that Hsp105’s nucleotide exchange activity plays a role in this process. This finding is consistent with the observation that overexpressing Hsp105 NE*-F or G*-F did not markedly stimulate infection (Fig 4C). As controls, overexpressing Hsc70, SGTA, or HspBP1 failed to diminish the number of cells harboring the BAP31-positive foci (Fig 4E, compare fifth and seventh bar graphs to second), in complete agreement with their inability to stimulate infection when overexpressed (Fig 4C). Hence, Hsp105 but not other chaperones promotes extraction of SV40 into the cytosol, a reaction that appears to impair the stable formation of the virus-containing foci.

While depleting Hsp105 enhanced foci formation (Fig 3E and 3F), we tested whether re-introducing Hsp105 under this condition can reverse the effect. Accordingly, Hsp105-depleted cells transfected with the siRNA-resistant *Hsp105* construct were infected and processed as before. Using this strategy, we found that while *Hsp105* knockdown increased the number of cells containing at least one BAP31-positive foci compared to control (Fig 4F, first two bar graphs), expressing WT Hsp105 under this condition reversed the effect (Fig 4F, second and third bar graphs). As controls, expressing either of the Hsp105 mutants, SGTA, or HspBP1 under the *Hsp105* knockdown condition did not promote loss of foci, whereas Hsc70 overexpression modestly impaired foci formation (Fig 4F, fourth to eight bar graphs). Hence Hsp105’s nucleotide exchange activity is essential for regulating foci formation. Because expressing WT but not mutant Hsp105 in Hsp105-depleted cells also restored infection (Fig 2I), these data further strengthen the functional connection between foci formation and productive infection.

### Hsp105 engages ER membrane-penetrating SV40 and promotes disassembly of the virus

**Fig 5:**
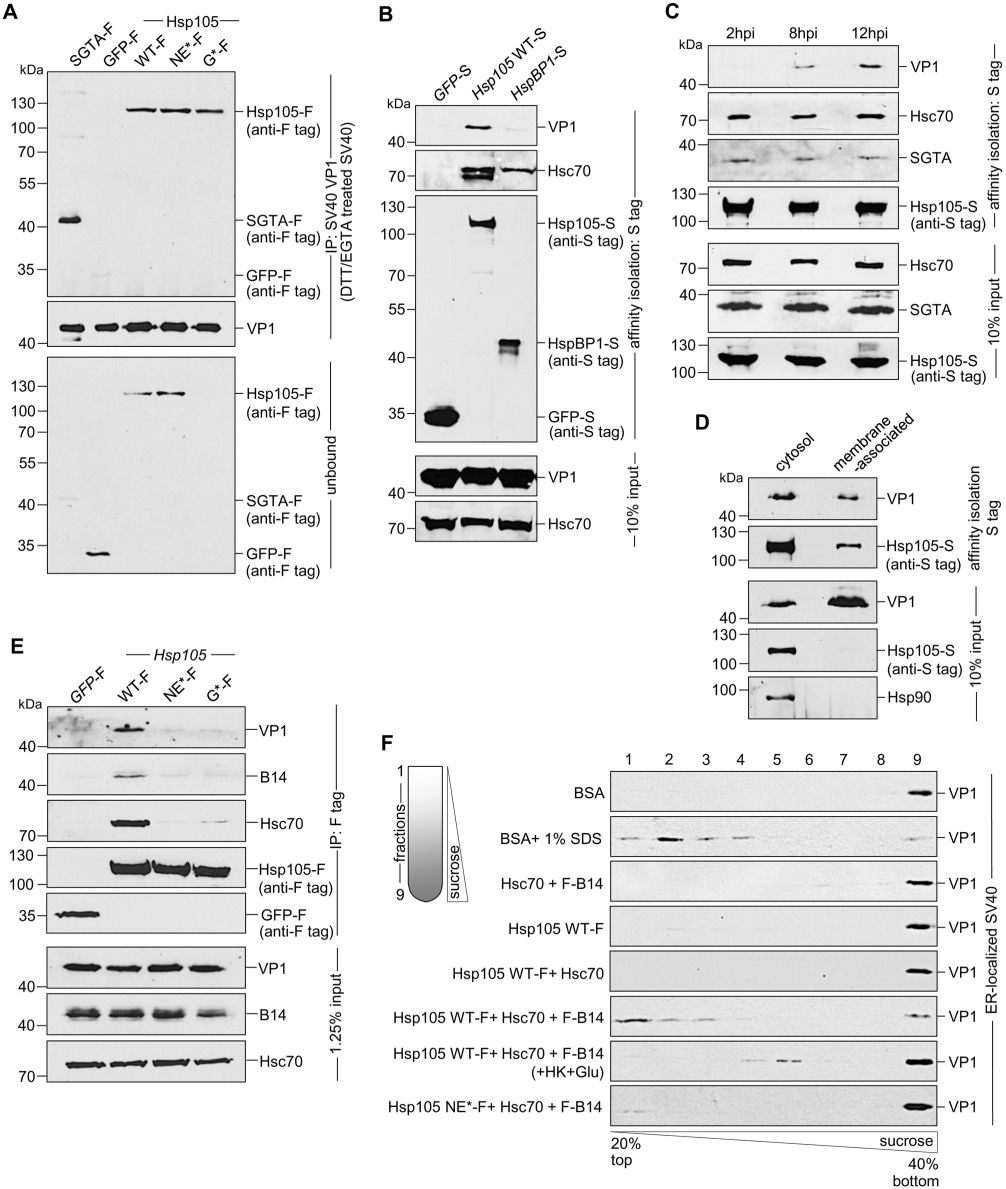
Hsp105 engages ER membrane-penetrating SV40 and promotes disassembly of the virus. **A**. The indicated purified protein in Fig 2E was incubated with DTT/EGTA-treated SV40. SV40 was immunoprecipitated from the sample and analyzed by immunoblotting using the indicated antibodies. **B**. CV-1 cells expressing the indicated S-tagged protein were infected with SV40 (MOI ~10). 12 hpi, S-tagged proteins affinity purified from WCE and immunoblot was performed with the indicated antibodies. **C**. As in (B), except cells were infected with SV40 for the indicated time. **D**. Cells expressing Hsp105 WT-S were infected with SV40 for 12 h, cross-linked with DSP, and fractionated to generate a cytosol and membrane fraction. Hsp105 WT-S was affinity isolated from each fraction, and the samples analyzed by immunoblotting with the indicated antibodies. **E**. As in (B), except the indicated F-tagged constructs were expressed in the cells. **F**. Triton X-100 extracted, ER-localized SV40 was incubated with the indicated purified protein(s), and subjected to discontinuous sucrose gradient centrifugation. Fractions were collected (as shown on the left side of the figure) from the top of the gradient and analyzed for the presence of SV40 by immunoblotting using VP1 antibodies.

Mechanistically, we envision that Hsp105 binds to SV40 at the ER-cytosol interface to extract the membrane-embedded virus into the cytosol. To determine if the Hsp105-SV40 interaction is direct, we incubated purified SV40 with purified proteins (Fig 2E); SGTA-F or GFP-F serves as a positive and negative control, respectively. In this experiment, SV40 was pretreated with the reducing agent dithiothreitol (DTT) and the calcium chelator EGTA to partially mimic conformational altered virus [36,40]. Importantly, when the virus was precipitated from the sample (Fig 5A, second panel), Hsp105 WT and mutant co-precipitated (Fig 5A, first panel, lanes 3–5); as expected, SGTA-F was pulled down but not GFP-F (Fig 5A, first panel, compare lane 1 to 2). Thus Hsp105 can interact with SV40 directly; because the Hsp105 mutants also bind to SV40 *in vitro*, they are unlikely globally misfolded.

To establish an interaction between Hsp105 and SV40 during cellular entry, cells expressing S tagged proteins were infected with SV40 in a synchronized manner for 12 h. The tagged proteins were affinity isolated and analyzed by immunoblot. While affinity isolation of either Hsp105 WT-S or HspBP1-S (but not GFP-S) pulled down Hsc70 (Fig 5B, second panel), only precipitation of Hsp105 WT-S pulled down SV40 (Fig 5B, first panel). This finding indicates that an Hsc70 complex containing Hsp105 engages the virus during infection. Because SV40 reaches the cytosol 6–8 hours post-infection (hpi) [37,39], we performed a time-course experiment to evaluate when Hsp105 associates with the virus. Cells expressing Hsp105 WT-S were infected in a synchronized manner for 2, 8, or 12 h. Hsp105 WT-S was affinity isolated and subjected to immunoblotting. We found that Hsp105 begins to engage the virus at 8 hpi, with the interaction increasing at 12 hpi (Fig 5C, first panel). The observation that SV40-Hsp105 binding is detected at approximately the same time point when the virus enters the cytosol suggests that Hsp105 engages the virus at the ER-cytosol interface.

As a second approach to test the idea that Hsp105 initiates its interaction with the virus at the ER membrane, we asked whether membrane-associated Hsp105 binds to SV40. To this end, Hsp105 WT-S was immunoprecipitated from the membrane (as well as the cytosol) fraction derived from infected cells. Interestingly, despite precipitating a lower level of Hsp105 WT-S from the membrane fraction when compared to the cytosol fraction (Fig 5D, second panel), no significant difference in the VP1 level from each fraction was co-precipitated (Fig 5D, first panel). This finding suggests that membrane-associated Hsp105 binds to SV40, consistent with the notion that this chaperone initiates its interaction with SV40 at the cytosolic surface of the ER membrane.

For a third approach to evaluate whether Hsp105 interacts with SV40 at the ER-cytosol interface, we tested if Hsp105 NE*-F and G*-F interact with SV40 and found that they cannot (Fig 5E, first panel, compare lanes 3 and 4 to 2). It is possible that these Hsp105 mutants are not targeted to the ER membrane because they cannot bind to transmembrane protein B14 via Hsc70 (Fig 5E, second and third panels, compare lanes 3 and 4 to 2). Alternatively, if B14 binds to Hsp105 directly, these Hsp105 mutants may fail to bind to B14 due to conformational changes resulting from the introduced mutations. Regardless, these results support the hypothesis that Hsp105 binds to SV40 at the ER-cytosol interface to extract the virus into the cytosol. Because Hsp105 NE*-F and G*-F interacts with SV40 *in vitro* (Fig 5A), their inability to bind to SV40 in cells (Fig 5E) is likely due to mis-localization away from the ER membrane.

How might Hsp105 engage and extract a large and intact viral particle embedded in the ER membrane [39] into the cytosol? One possibility is that Hsp105, in concert with the Hsc70 complex, disassembles the membrane-penetrating virus. This would destabilize the structural integrity of the membrane-embedded virus, enabling it to be released into the cytosol more efficiently. To test this, a sucrose gradient sedimentation assay modified slightly from previous studies was used [36,39,55]. In this assay, Triton X-100 extracted ER-localized SV40 (including membrane-penetrating virus) was incubated with ATP and the indicated purified proteins (Fig 2E). The samples were layered on a discontinuous sucrose gradient and centrifuged (Fig 5F). Individual fractions were collected and subjected to immunoblotting with anti-VP1 antibodies. In this fractionation procedure, any VP1 liberated from disassembled viral particles should appear in the top fractions corresponding to light sucrose density, whereas dense viral particles should appear in the bottom fractions with a denser sucrose concentration. Incubation with BSA retained viral VP1 signal at the dense bottom fraction (Fig 5F, first panel), confirming that ER-localized virus is intact as previously reported [39]. By contrast, addition of SDS caused a significant pool of VP1 to shift to the top fractions (Fig 5F, second panel), reflecting the ability of this detergent to chemically disassemble the virus [36]. Strikingly, whereas addition of Hsc70+B14, Hsp105 alone, or Hsp105+Hsc70 did not alter the fractionation pattern when compared to incubation with BSA (Fig 5F, third-fifth panels), inclusion of all three components (Hsp105 WT-F+Hsc70+F-B14) caused a significant portion of VP1 signal to distribute to the top fractions (Fig 5F, sixth panel). These findings indicate that Hsp105 must operate in concert with B14 and Hsc70 to stimulate disassembly of ER-localized virus. The distribution of VP1 signal to the top fraction represents VP1 pentamers liberated from disassembled viral particle, consistent with previous reports. The Hsp105-driven disassembly reaction is energy-dependent as addition of hexokinase and glucose (used to deplete ATP) to the Hsp105+Hsc70+B14 sample reduced the appearance of the disassembled virus in the top fractions (Fig 5F, seventh panel); a low level of virus nonetheless appeared in the middle fractions (fractions 4 and 5), possibly reflecting the use of non-hydrolyzed ATP that generated a partially uncoated SV40 intermediate. When the Hsp105 NE* mutant was incubated with the Hsc70-B14 complex, minimal virus disassembly was observed (Fig 5F, eighth panel), implicating the nucleotide exchange activity of Hsp105 as crucial in the disassembly reaction. Together, these findings suggest that Hsp105 binds to the membrane-penetrating virus at the ER-cytosol interface, and can potentially promote disassembly of the viral particle to facilitate extraction into the cytosol.

## Discussion

**Fig 6:**
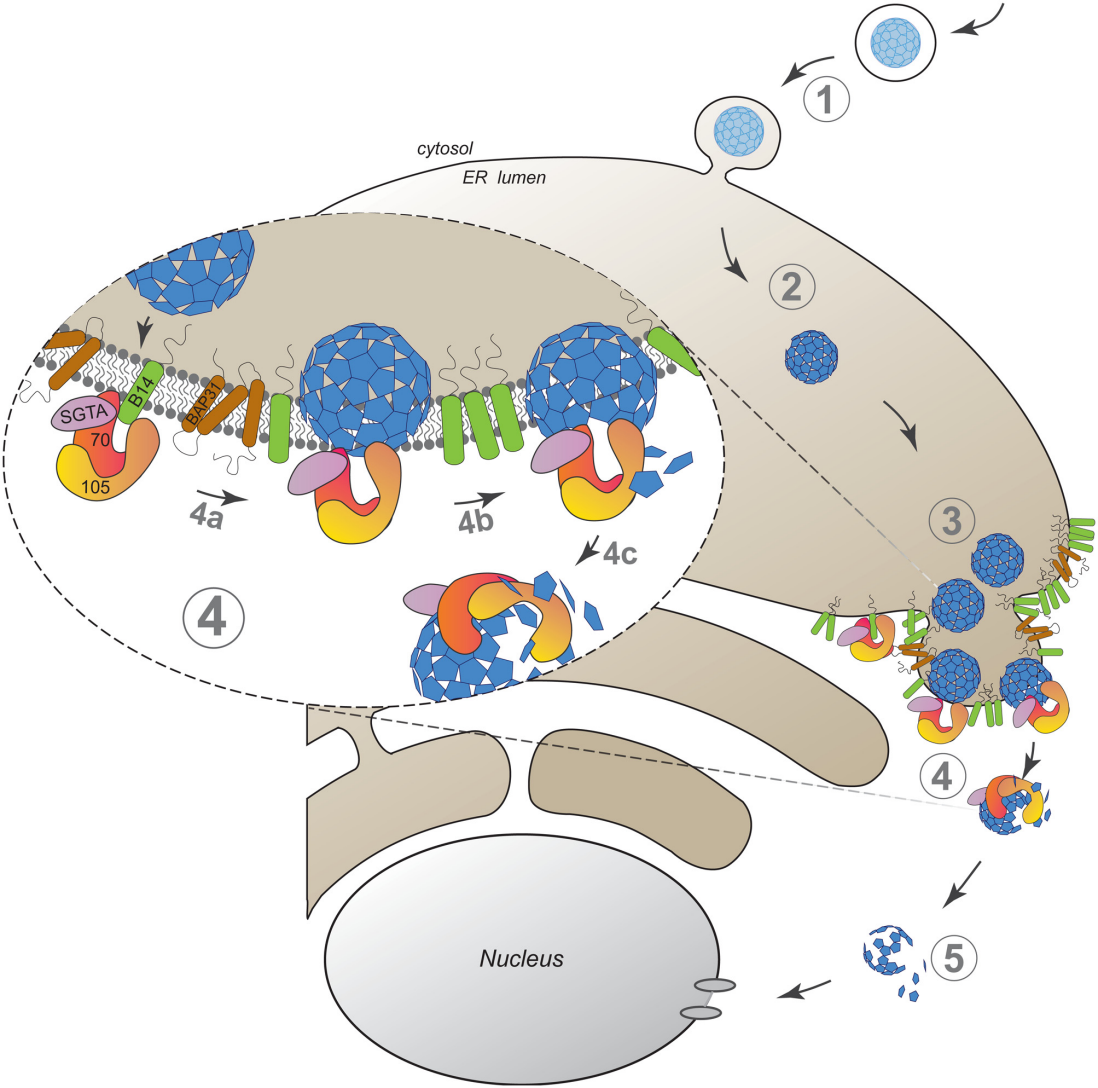
A model depicting Hsp105-dependent extraction of SV40 from the ER into the cytosol. SV40 infection is initiated when the virus traffics from the cell surface to the ER (step 1). In the ER, specific ER-resident isomerase and reductase induce conformational changes to the virus, generating a hydrophobic particle (step 2). The hydrophobic virus then binds to and integrates into the ER membrane where SV40 accumulates into discrete foci (step 3). We envision that Hsp105, anchored to the membrane-bound J-protein B14 directly or indirectly via Hsc70/Hsp70, binds to the membrane-penetrating virus (step 4a, see insert). Continuous cycles of binding-release of SV40 by Hsp105-Hsc70 initiates the extraction process, a step that may be coupled to disassembly of the membrane-embedded viral particle (step 4b). Extraction is completed when SV40 is fully released into the cytosol (step 4c). Upon cytosol arrival, a partially disassembled viral particle intermediate mobilizes into the nucleus to stimulate infection (step 5).

Our findings here establish an unanticipated role of an Hsp110 family member in driving membrane translocation of a viral particle. Specifically, we demonstrate that SV40 co-opts Hsp105 to cross the ER membrane and reach the cytosol in order to promote infection. SV40 infection begins when it traffics from the cell surface to the ER (Fig 6, step 1). In the ER, specific ER-resident isomerase and reductase act on the viral particle, imparting conformational changes to expose the hidden VP2/VP3 which generates a hydrophobic particle (Fig 6, step 2). The structurally altered virus then binds to and integrates into the ER membrane where SV40 accumulates into discrete foci (Fig 6, step 3). In our model, Hsp105, anchored to the membrane J-protein B14 directly or indirectly through Hsc70/Hsp70, binds to the membrane-penetrating virus (Fig 6, step 4a, see insert). Next, we hypothesize that iterative binding-release of SV40 by Hsp105-Hsc70 initiates the extraction process, a step that may involve disassembly of the membrane-embedded viral particle (step 4b). Extraction is completed when SV40 is fully released into the cytosol (step 4c). Upon cytosol arrival, a sub-viral particle intermediate is likely further processed to transport into the nucleus to cause infection (Fig 6, step 5).

The discovery that an Hsp110 family member is responsible for a membrane translocation event might appear surprising given that this chaperone family has generally been studied in the context of its chaperone activity during protein quality control in the cytosol [18,56,57]. However, as our biochemical analyses suggest that a pool of Hsp105 is localized to the ER via binding to the transmembrane J-protein B14, Hsp105 might control ER-associated protein quality control. The most well characterized ER protein quality control process is called ER-associated degradation (ERAD), a pathway that in fact involves translocation of a misfolded ER substrate across the ER membrane to the cytosol via an elaborate machinery where the substrate is degraded by the ubiquitin-dependent proteasomal system [58,59]. ER-to-cytosol translocation of SV40 is reminiscent of the fate of misfolded proteins in the ERAD pathway. Although a myriad of cellular factors sub-serving a defined function during ERAD have been identified [58], including Hsp105’s membrane-binding partner B14 and the related B12 [44,60–62], a role of Hsp105 itself in ERAD remains unclear [18,63].

Recent findings revealed that elements of the ERAD machinery are hijacked by toxic agents including viruses and bacterial toxins [36,39,64]. During host entry, these toxic agents are thought to disguise as misfolded proteins, co-opting components of the ERAD machinery in the ER to gain access to the cytosol. And by evading the cytosolic proteasome [65], these toxic agents are able to avoid a degradative fate to continue their cellular journey. SV40 serves as a salient example of a virus that hijacks the ERAD pathway during entry. While this virus indeed exploits many ERAD components to prepare it for ER-to-cytosol translocation [36,37,40,51,53], how SV40 is ultimately ejected into the cytosol from the ER membrane represents its most enigmatic entry step. This is especially salient given that the cytosolic p97 ATPase that normally extracts typical cellular misfolded ERAD substrates to the cytosol [66] is not used to extract the membrane-embedded SV40 into the cytosol, and that the Hrd1 E3 ubiquitin ligase linking p97 to the ER membrane also appears not to subserve any role during viral translocation [37]. Hence, the identity of the extraction machinery responsible for SV40 cytosol entry is unknown. Interestingly, cholera toxin also reaches the cytosol from the ER using a p97-independent process [67,68].

In this context, the loss- and gain-of-function data presented in this study strongly argue that Hsp105 plays a major role in extracting SV40 into the cytosol from the ER membrane. We found that this step requires the nucleotide exchange activity of Hsp105, suggesting that Hsp105 must act in concert with Hsc70 to drive the release of the viral particle into the cytosol. Our previous analyses demonstrated that SV40 penetrates the ER membrane as a large and intact viral particle [39]. A separate study suggested that SV40 undergoes modest uncoating in the ER without experiencing massive disassembly, morphing from its native 45 nm to 34 nm when it is in the ER [37]. How then might the Hsp105-Hsc70-SGTA-B14 complex generate the force necessary to eject this large membrane-embedded viral species into the cytosol? As stated, Hsp110 in conjunction with Hsc70 and a J-protein can disaggregate an aggregated model substrate [7,22–25]. Paralleling this proposed disaggregation activity, we found that purified Hsp105, Hsc70, and B14 drive disassembly of the ER-localized SV40. SV40 in fact disassembles in the cytosol during the infection course [39,69]. While a previous *in vitro* study demonstrated that eukaryotic Hsc70 and J-protein can uncoat native murine PyV when incubated overnight [55], whether addition of a mammalian NEF to this system can stimulate the rate of uncoating requires further investigation.

As our data demonstrate that the Hsc70-coupled Hsp105 machinery extracts SV40 into the cytosol, we speculate that the physical force generated by this machinery disassembles the membrane-penetrating virus, thereby destabilizing the viral particle to enable more efficient extraction into the cytosol. If this is the case, this disassembly may involve disruption of the VP1 C-terminal arm that normally stabilizes SV40 interpentamer interactions [30,31]. This disruption, coupled with reduction of any remaining interpentamer disulfide bonds in SV40 by the highly reducing cytosolic environment, should lead to formation of disassembled VP1 pentamers. Interestingly, the use of the Hsc70-associated machinery to disassemble the large and intact ER-localized viral particle is reminiscent of the disassembly of the approximate 150–200 nm clathrin basket by the Hsc70 machinery [70–72].

In infected cells, SV40 accumulates in foci within the ER postulated to represent the cytosol entry site [37,43,54]. Our imaging analyses performed under Hsp105 loss- and gain-of-function conditions further support this idea. Specifically, overexpressing Hsp105 impaired the formation of the virus-containing foci leading to increased cytosol extraction, while down-regulating Hsp105 enhanced foci formation thereby blocking cytosol arrival. The correlation between Hsp105’s capacity to promote loss of the SV40-containing foci and cytosol arrival of the virus strengthens the notion that the foci represent a portal for viral cytosol entry.

While chaperones are vital for maintaining proper protein folding, they are equally crucial in driving protein translocation across biological membranes [73]. This is arguably most evident in protein translocation across the ER membrane. For instance, during post-translational forward translocation where nascent polypeptides are translocated from the cytosol into the ER, the ER-resident Hsc70 chaperone BiP “pulls” substrates into the lumen via a Brownian ratcheting mechanism [74], whereas during ERAD when typical misfolded proteins are translocated from the ER back to the cytosol, the cytosolic p97 “pulls” the substrates into cytosol [66]. Our discovery that an Hsp110 family member can “pull” a large protein complex (a viral particle) from the ER into the cytosol suggests that this chaperone family member may also exert an important role during ERAD. Clearly future experiments will be required to expand on this exciting possibility.

## Methods

### Reagents

CV-1, BSC-1, COS-7 and HEK 293T cells (ATCC) were grown in complete DMEM (cDMEM; containing 10% fetal bovine serum, 10 U/ml penicillin, and 10 μg/ml streptomycin; Gibco, Grand Island, NY). Opti-MEM and 0.25% trypsin-EDTA were purchased from Gibco. Sources of the other reagents are as follows: dithiobis(succinimidyl proprionate) (DSP; Thermo, Rockford, IL), digitonin and S tag protein-conjugated agarose beads (EMD Millipore, San Diego, CA), protein A conjugated agarose beads and protein G conjugated magnetic beads (Life Technologies, Carlsbad, CA), anti-FLAG M2 antibody-conjugated agarose beads, phenylmethanesulfonylfluoride (PMSF) and Triton X-100 (Sigma, St. Louis, MO).

### Preparation of SV40

SV40 was purified using the OptiPrep (60% stock solution of iodixanol in water; Sigma) gradient centrifugation method described previously in [39]. Briefly, viral genome transfected CV-1 cells were lysed in a buffer containing 50 mM Hepes pH 7.5, 150 mM NaCl and 0.5% Brij 58 for 30 min on ice, and the supernatant was collected after centrifugation at 20,000x g for 10 min. The supernatant was placed on top of a discontinuous OptiPrep gradient of 20% and 40%, and centrifuged at 49,500 rpm for 2 h at 4°C in an SW55Ti rotor (Beckman Coulter, Indianapolis, IN). A white interface formed between 20% and 40% OptiPrep was collected, and aliquots were stored at -80°C for future use. Purified BKV and antibody against BKV large TAg (pAB416) were generous gifts from Dr. Michael Imperiale (University of Michigan).

### Plasmids

All the plasmids used in this study contain pcDNA3.1 (-) as vector backbone and the sources of these plasmids are: F-*B14*, F-*B14* H136Q, *SGTA*-F and *GFP*-F [43]. Protein tags (S- or F-) at the N- or C-terminus are depicted as prefix or suffix, respectively. *Hsp105* WT-S was generated from the plasmid pET28a-*Hsp105* (a gift from Dr. Eileen Lafer, University of Texas). The siRNA-resistant *Hsp105* WT was generated by introducing silent mutations (underlined 343-GAGCAGATAACAGCCATGTTGTTGA-367) using two rounds of PCR and the FLAG tag was introduced to obtained *Hsp105* WT-F. The mutant *Hsp105* N636Y/E639A (NE*) and G9L (G*) were created based on [24,51] by overlapping PCR using siRNA resistant *Hsp105* WT-F. The gene products were then inserted into the vector backbone with FLAG tag. The *HspBP1*-S and *Hsc70*-S were amplified from a HEK 293T cDNA library and inserted into a vector with an S tag. The list of primers used to generate all the above plasmids is given in Table 2.

### DNA transfection

For overexpression studies, 50% confluent CV-1 cells in 6 cm, 10 cm or 15 cm plates were transfected with plasmid using the FuGENE HD (Promega, Madison, WI) transfection reagent at a ratio of 1:4 (plasmid to transfection reagent; w/v). Cells were allowed to express the protein for at least 24 h before experiments. For COS-7 cells in 6 cm plate, polyethylenimine (PEI; Polysciences, Warrington, PA) was used as the transfection reagent.

### siRNA transfection

For the knockdown of *Hsp105*, *SGTA* and *HspBP1*, custom stealth siRNAs were generated and purchased from Invitrogen (Carlsbad, CA). *Hsp105* siRNA #1 target sequence was adapted from [18], and *HspBP1* siRNA target sequence was adapted from [75]. *Hsc70* siRNA was purchased from Dharmacon (Lafayette, CO; catalogue: J-017609-08). The list of siRNAs used in this study is given in Table 3.

For microscopy experiments, 2x10^4^ CV-1 cells were seeded on coverslips in 12-well plates and for all other experiments, 2x10^5^ cells were seeded in 6 cm plates and were reverse transfected with 10 nM *Hsp105* #1 or 12.5 nM *Hsp105* #2 siRNA using Lipofectamine RNAiMAX reagent (Invitrogen) for at least 24 h. The ratio of siRNA to transfection reagent was maintained at 1:4 v/v. For knockdown followed by rescue experiments, 24 h post-siRNA transfection, cells were washed with cDMEM and transfected with siRNA resistant plasmids. Cells are allowed to express the protein for at least 24 h before experimentation.

### *XBP1* splicing assay

Detection of *XBP1* splicing was performed as described previously [76]. The primers used were listed in Table 4.

### Immunoprecipitation and affinity purification

Transfected cells were harvested using trypsin and cell pellets were washed three times with cold phosphate buffered saline (PBS, Gibco). Washed cells were lysed in TSEp buffer (50 mM Tris-Cl pH 7.5, 150 mM NaCl, 1 mM EDTA and 1 mM PMSF) with 0.2% digitonin at 4°C for 10 min. For protein crosslinking studies, harvested cells were incubated with freshly prepared 2 mM DSP (dithiobis(succinimidyl proprionate)) for 30 min at room temperature with intermittent shaking. This membrane permeable, amine-reactive, and thiol-cleavable crosslinker was used to stabilize transient or weak protein-protein interactions. Excess cross-linker was quenched with 200 mM Tris pH 7.5. Cells were then lysed with 1% Triton X-100 in TSEp buffer at 4°C for 10 min. Cell lysate were clarified by centrifugation at 20,000x g for 10 min at 4°C. The resulting supernatant was immunoprecipitated with anti-FLAG conjugated agarose beads or affinity purified with S tag protein-conjugated agarose beads for 2 h at 4°C. Samples were eluted with 1x SDS sample buffer with 1.25% β-mercaptoethanol (Sigma) and boiled for 5 min at 95°C before subjected to SDS-PAGE and immunoblotting.

### Immunopurification and identification of B14 binding partners

Flp-In 293 T-Rex cells (Invitrogen) transfected pcDNA5-*B14*-3xF was used to immunopurify B14-3xF as in [43]. Briefly, cells selected in media containing blasticidin and hygromycin (Invitrogen) were induced overnight with freshly prepared 5 ng/ml tetracycline (Sigma) to express B14-3xF to near endogenous level. 10 μM ganglioside GM1 (Matreya, Pleasant Gap, PA) was supplemented to the culture media prior to infection. Next day, near confluent cells were infected with SV40 (MOI ~50) for 16 h. HEK 293T cells were used as negative control. Post infection, cells were harvested with cold PBS and centrifuged at 500x g for 5 min. Cells pellets were lysed in 2.5 ml buffer containing 0.1% digitonin in TSEp buffer for 30 min in ice. Lysate was centrifuged at 20,000x g for 15 min and the supernatant was incubated with anti-FLAG agarose conjugated beads for 2 h at 4°C. Beads were washed three times with TSEp buffer, and the proteins were eluted twice using 3x FLAG peptide (200 μl, 0.25 mg/ml in PBS) (Sigma) for 1 h at 4°C. Eluents were concentrated using centrifugal filters (Amicon Ultra 3K, Cork, Ireland), and the concentrated samples were separated on SDS-PAGE and either visualized by silver staining (Invitrogen) or immunoblotted. For mass spectrometry analysis, protein bands were excised from the silver stained gel, and analyzed at Taplin Biological Mass Spectrometry Facility (Harvard Medical School). The data obtained were processed based on number of unique peptides identified, percentage sequence coverage, and the observed molecular weight.

### Semi-permeabilized cytosol arrival assay

Cells were prechilled at 4°C for 20 min before infecting with SV40 (MOI ~5) for 1 h at 4°C. Cells were washed once with cold cDMEM, warm cDMEM was added, and cells were incubated for 12 h at 37°C. Post infection, cells were lysed in HNp buffer (50 mM Hepes pH 7.5, 150 mM NaCl and 1 mM PMSF) containing 0.1% digitonin at 4°C for 10 min, and separated into supernatant (cytosol) and pellet (membrane) fractions by centrifugation at 20,000x g for 10 min at 4°C. To isolate ER-localized SV40, the pellet fraction was further treated with HNp buffer containing 1% Triton X-100 for 10 min at 4°C and centrifuged at 20,000x g for 10 min at 4°C. The fractions were then dissolved in 1x SDS sample buffer containing 1.25% β-mercaptoethanol, and boiled for 5 min at 95°C before immunoblotting. To assess cytosol arrival of cholera toxin A1 (CTA1) subunit, CV-1 cells were treated with 10 nM CT (EMD Millipore) for 90 min. Cells were harvested and fractionated as above.

### Preparation of purified proteins

Purified Hsc70 was purchased from StressMarq Biosciences (Victoria, Canada). For purification of F-B14, SGTA-F, GFP-F, Hsp105 WT-F, Hsp105 NE*-F and Hsp105 G*-F proteins, confluent HEK 293T cells in 15 cm plates were transfected with pcDNA3.1 (-)-FLAG tag plasmids using 1:4 ratio of DNA to PEI transfection reagent (w/w). After transfection for 24–48 h, cells were washed three times with PBS and harvested using trypsin. Cell pellets were lysed in HNp buffer containing 1% Triton X-100 for 20 min at 4°C. Cell lysates were cleared by centrifugation at 20,000x g for 10 min at 4°C and the supernatant was incubated with 25 μl of anti-FLAG M2 agarose conjugated beads for 2 h at 4°C. Beads were washed three times with HNp buffer and incubated with HKM buffer (20 mM Hepes pH 7.5, 50 mM KCl, 2 mM MgCl_2_) containing 0.1% Triton X-100 and 2 mM ATP for 30 min at room temperature. Experiments involving F-B14 contains 0.1% Triton X-100 throughout. Bound proteins were eluted twice using FLAG peptide (100 μl, 0.25 mg/ml; Sigma) for 30 min at 4°C. Eluents were concentrated using centrifugal filters (Amicon Ultra 3K) and the concentrated samples were separated on SDS-PAGE and visualized using Brilliant Blue R250 (Thermo Fisher) staining.

### *In vitro* binding assay

OptiPrep purified SV40 was treated with 3 mM DTT and 10 mM EGTA for 45 min at 37°C. The reaction was passed through Micro biospin P-30 Tri-chromatography columns (Biorad, Hercules, CA) and SV40 was eluted using PBS. Approximately 250 ng of SV40 was incubated with 0.2–0.5 μM of purified FLAG tag proteins. The reaction was made up to 50 μl with PBS and incubated for 1 h at 25°C, followed by overnight incubation with anti-VP1 at 4°C using an end-over-end rotor. The reaction was then incubated with 10 μl of protein G conjugated magnetic beads for 2 h at 4°C. Unbound fraction was collected and the beads were washed three times with PBS. Bound proteins were eluted with 1x SDS sample buffer with 1.25% β-mercaptoethanol, and boiled for 5 min at 95°C before immunoblotting.

### ATP binding assay

Purified FLAG tag proteins (0.2–0.5 μM) were initially incubated with HKM buffer for 30 min at room temperature and then incubated with 10 μl of ATP-agarose beads (Innova Biosciences, Cambridge, UK) for 30 min at room temperature with gentle agitation. Unbound fraction was collected and the beads were washed three times with HKM buffer. Bound proteins were eluted with 1x SDS sample buffer with 1.25% β-mercaptoethanol and boiled for 5 min at 95°C before immunoblotting.

### Nucleotide release assay

An assay, based on [77] and [78], was developed to test the nucleotide exchange activity of WT and mutant Hsp105 against Hsc70. Hsc70 (3 μM) was incubated with 50 μCi of [α-^32^P] ATP (3000 Ci/mmol; Perkin Elmer, Waltham, MA) in a final volume of 25 μl (50 μCi is equivalent to 0.66 μM ATP in this reaction) at 37°C for 30 min to form [α-^32^P] ADP Hsc70, and the sample subjected to a spin gel filtration column (GE Healthcare, Cleveland, OH) to remove the free nucleotides. [α-^32^P] ADP Hsc70 was incubated with 0.3 μM of FLAG tag proteins (GFP-F, Hsp105 WT-F, Hsp105 NE*-F or Hsp105 G*-F) in 23 μl of a buffer containing 20 mM Hepes (pH 7.5), 50 mM KCl and 0.1% Triton X-100 at 23°C for 20 min. Following incubation, each reaction was mixed with unlabeled 0.3 μM ATP and 2 mM MgCl_2_ and further incubated at 23°C for 1 min. After removal of free nucleotides using another spin gel filtration column, 2 μl of the reaction was spotted onto a PEI cellulose TLC plate (Sigma). The plates were developed in 0.6 M KH_2_PO_4_ pH 3.4 as the solvent system. Once dried, the plates are developed by exposing on a photographic film.

### SV40 disassembly assay

ER-localized SV40 was incubated in HKM buffer containing 2 mM ATP and 0.1% Triton X-100 with chaperones at the following concentrations: Hsc70 (~2 μM), F-B14 (1 μM), Hsp105 WT-F, or Hsp105 NE*-F (0.4 μM). BSA (1 μM) with or without 1% SDS was used as a control. Total volume of 20 μl was maintained throughout the reaction. Reaction was incubated at 37°C for 1 h and the sample placed on top of a discontinuous sucrose gradient consisting of 20%, 30% and 40% sucrose. Samples were centrifuged at 50,000 rpm in a TLA100 rotor (Beckman Coulter) for 30 min at 4°C, and individual fractions collected from the top of the gradient. Samples were then subjected to immunoblotting using antibodies against VP1.

### Immunofluorescence microscopy

CV-1 cells were grown and transfected on sterile cover slips. Cells were infected with MOI ~0.5 (for TAg expression studies) or MOI 20–30 (for foci formation studies) for 24 h and 16 h, respectively. Infected cells were fixed with 1% formaldehyde for 15 min at room temperature followed by permeabilization with 0.2% Triton X-100 in PBS for 5 min. Cells are then covered for 15 min with blocking buffer containing 5% milk in TBST (Tris buffered saline with 0.02% Tween 20). Cells were then immunostained with primary antibody diluted in blocking buffer for 1 h at room temperature and then washed five times with blocking buffer. Cells were then incubated with fluorescence dye conjugated secondary antibody for 30 min and then washed three times with blocking buffer, PBS, and water before air drying and mounting on glass slides (Fisher) using ProLong gold (Invitrogen) with or without DAPI (Molecular Probes, Eugene, OR). Slides were then allowed to dry in dark at room temperature for at least 12 h before imaging. Images were taken using an inverted epifluorescence microscope (Nikon Eclipse TE2000-E, Melville, NY) equipped with 40x, 60x and 100x 1.40 NA objectives and standard DAPI (blue), FITC (green) and TRITC (red) filter cubes. Images were processed using the ImageJ software version 1.48i (NIH). Cells were counted either under a microscope with an eyepiece or with the help of ImageJ program (Plugin: Cell counter). In each experiment, >1000 cells were scored for TAg positive cells or >100 were scored for BAP31 foci positive cells, in the indicated channel. Foci intensity and area profile are plotted with the assistance of ImageJ program (Plugin: Surface plot).

### Statistical analysis

Data obtained from at least three independent experiments were combined together for statistical analyses. Results were analyzed using Student’s *t* test. Data are plotted using GraphPad Prism software, version 5.0b. Data are represented as the mean values and error bar represents standard deviation (SD) (*n* ≥3) where indicated. * *p* < 0.05, ** *p* < 0.01, *** *p* < 0.001 were considered to be significant unless otherwise noted.

## Supporting Information

S1 Fig Hsp105 depletion reduces SV40 infection in BSC-1 cells, related to Fig 2. **A**. BSC-1 cells were transfected with ctrl or Hsp105 #1 siRNA. The resulting WCEs were immunoblotted with an antibody against Hsp105. **B**. As in Fig 2B, except BSC-1 cells were used.
(TIF)

S2 Fig SV40-induced foci colocalizes with specific ER membrane proteins, related to Figs 3 and 4. CV-1 cells infected with SV40 (MOI ~30) for 16 h were fixed, stained for SV40 VP1 and VP2/3, or the ER membrane proteins BAP31, B14, and B12, and imaged. BAP31 staining is used to mark the foci in all of the experiments, with the merged channels shown on the left. White arrowheads indicate foci. Scale bar, 10 μm.
(TIF)

S3 Fig SV40-induced foci are likely composed of multimeric viral particles, related to Figs 3 and 4. CV-1 cells infected with SV40 (MOI ~30) for 16 h were fixed and stained for VP2/3 and BAP31. Merged image is shown on the left. Intensity of the foci in the boxed area was analyzed by using ImageJ software, and the values are plotted as intensity versus dimension. Four different examples of the virus-induced foci are shown.
(TIF)
