# Supplementary figures and images for: A Non-enveloped Virus Hijacks Host Disaggregation Machinery to Translocate across the Endoplasmic Reticulum Membrane
Source: PLoS Pathog. 2015 Aug 5;11(8):e1005086. doi: 10.1371/journal.ppat.1005086 (PMC4526233; doi:10.1371/journal.ppat.1005086)

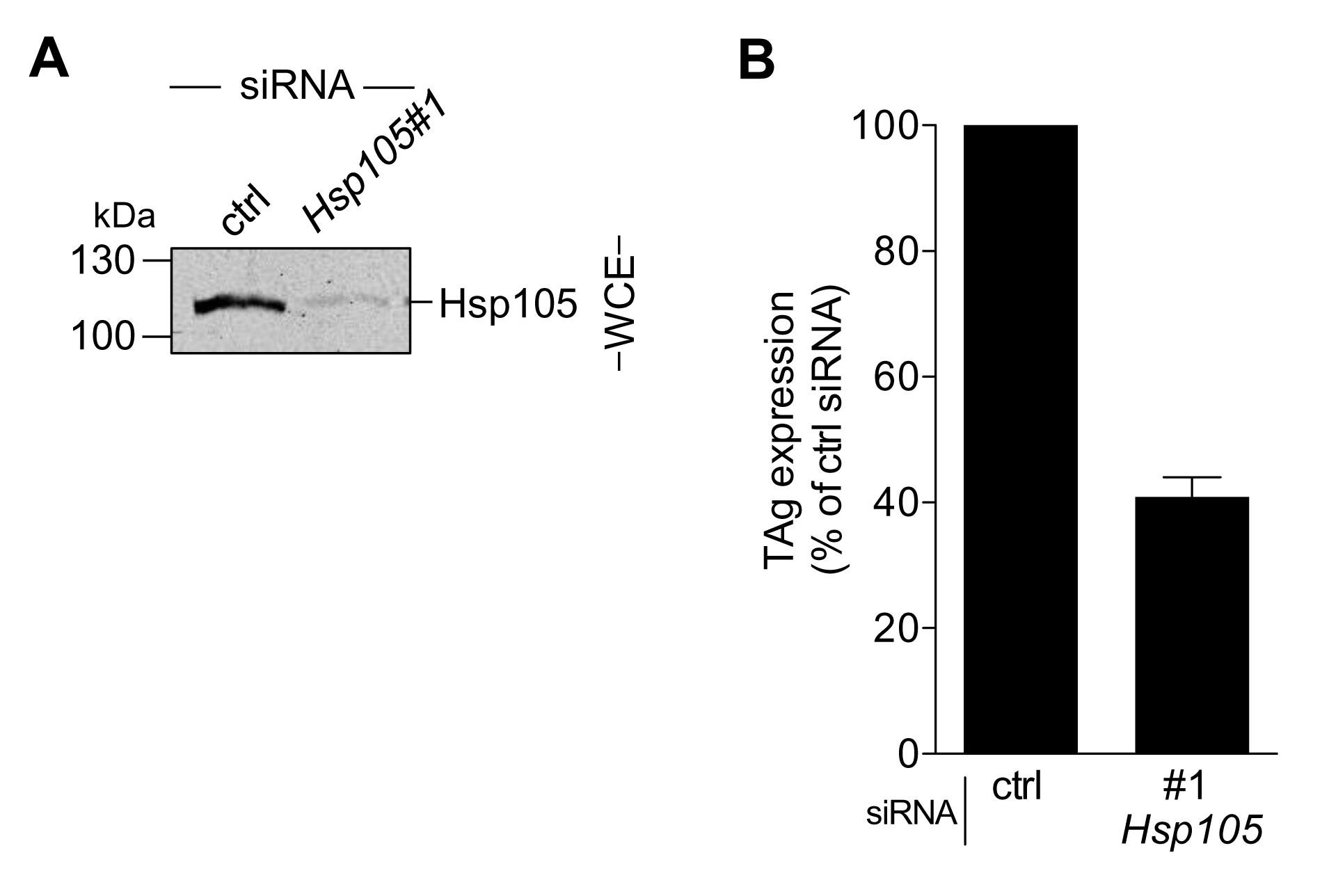

Supplement: S1 Fig — A. BSC-1 cells were transfected with ctrl or Hsp105 #1 siRNA. The resulting WCEs were immunoblotted with an antibody against Hsp105. B. As in Fig 2B, except BSC-1 cells were used. (TIF) [file ppat.1005086.s001.tif]

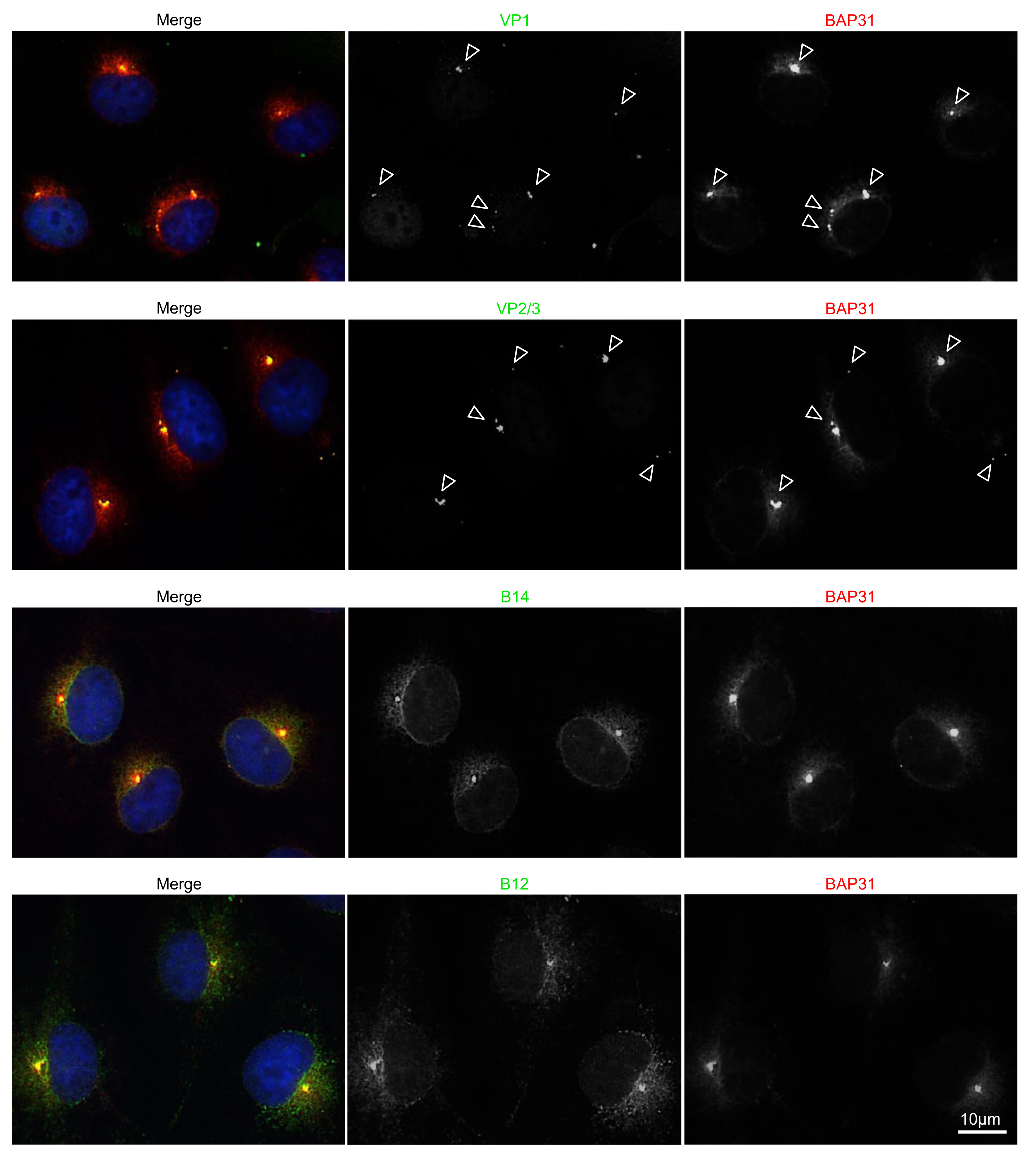

Supplement: S2 Fig — CV-1 cells infected with SV40 (MOI ~30) for 16 h were fixed, stained for SV40 VP1 and VP2/3, or the ER membrane proteins BAP31, B14, and B12, and imaged. BAP31 staining is used to mark the foci in all of the experiments, with the merged channels shown on the left. White arrowheads indicate foci. Scale bar, 10 μm. (TIF) [file ppat.1005086.s002.tif]

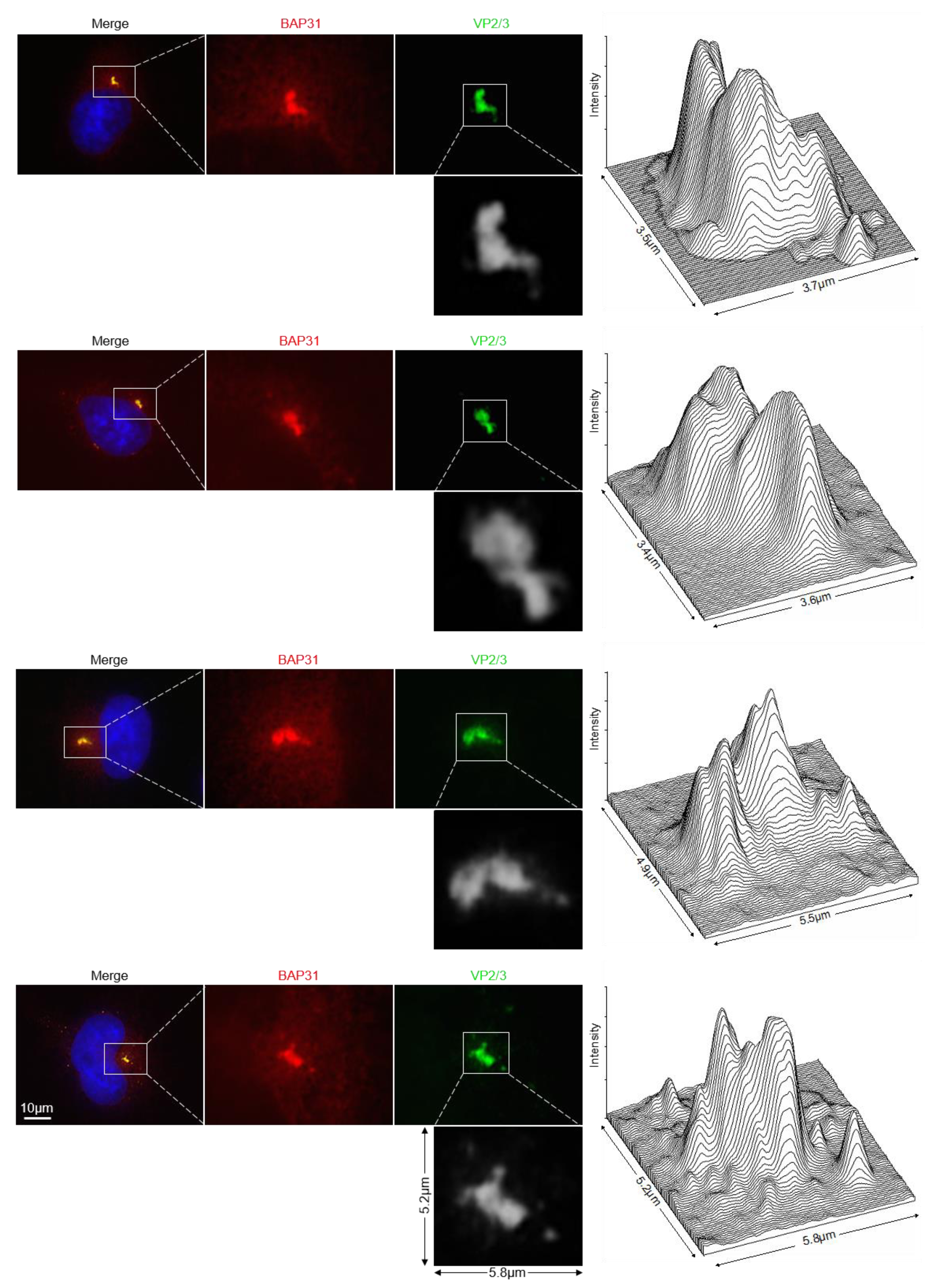

Supplement: S3 Fig — CV-1 cells infected with SV40 (MOI ~30) for 16 h were fixed and stained for VP2/3 and BAP31. Merged image is shown on the left. Intensity of the foci in the boxed area was analyzed by using ImageJ software, and the values are plotted as intensity versus dimension. Four different examples of the virus-induced foci are shown. (TIF) [file ppat.1005086.s003.tif]
